# Surface engineered NiFe_2_O_4_/SnO_2_/CeO_2_ ternary heterojunction for dual applications in photocatalytic water treatment and supercapacitors

**DOI:** 10.1039/d5ra07855e

**Published:** 2025-11-13

**Authors:** Adewumi O. Oluwole, Samba Sarr, Tunde L. Yusuf, Shepherd M. Tichapondwa, Michael O. Daramola, Samuel A. Iwarere

**Affiliations:** a Department of Chemical Engineering, Faculty of Engineering, Built Environment and Information Technology, University of Pretoria Hatfield Pretoria 0002 South Africa omayor8@gmail.com samuel.iwarere@up.ac.za +27-12-420-3092; b Department of Chemistry, Faculty of Natural and Agricultural Sciences, University of Pretoria Hatfield Pretoria 0002 South Africa

## Abstract

Surface-engineered ternary NiFe_2_O_4_/SnO_2_/CeO_2_ (NFO/SnO_2_/CeO_2_) heterojunctions were synthesised through a facile hydrothermal route to deliver dual-function material for visible-light photocatalysis and electrochemical energy storage. Comprehensive structural, optical and morphological characterisation confirmed intimate interfacial coupling between the three oxides, a mesoporous architecture (72.99 m^2^ g^−1^) and a narrowed band gap of 1.53 eV, all of which promote efficient charge separation and extended visible-light harvesting. Under visible light irradiation, the optimised composite achieved 97.92% degradation of tetracycline within 60 min, five to nineteen-fold higher than the pristine or binary counterparts, following pseudo-first-order kinetics (*k* = 0.04018 min^−1^). Reactive-species quenching identified ˙O_2_^−^ and ˙OH radicals as the dominant oxidants, and the catalyst retained >85% activity after five cycles, demonstrating excellent photostability. This same material delivered a high specific capacity of 106.7 mA h g^−1^ at 1 A g^−1^, a coulombic efficiency of 98.7% and 74.5% capacity retention over 4000 charge–discharge cycles in 6 M KOH, outperforming the individual oxides owing to synergistic redox behaviour and rapid ion diffusion across the heterointerfaces. This work provides mechanistic insights and a scalable synthesis platform for designing next-generation multifunctional oxides integrating photocatalytic and supercapacitive functions within a single, magnetically recoverable nanocomposite for environmental and energy applications.

## Abstract 2

Surface-engineered ternary NiFe_2_O_4_/SnO_2_/CeO_2_ (NFO/SnO_2_/CeO_2_) heterojunctions were synthesised through a facile hydrothermal route to deliver dual-function material for visible-light photocatalysis and electrochemical energy storage.

## Graphical abstract

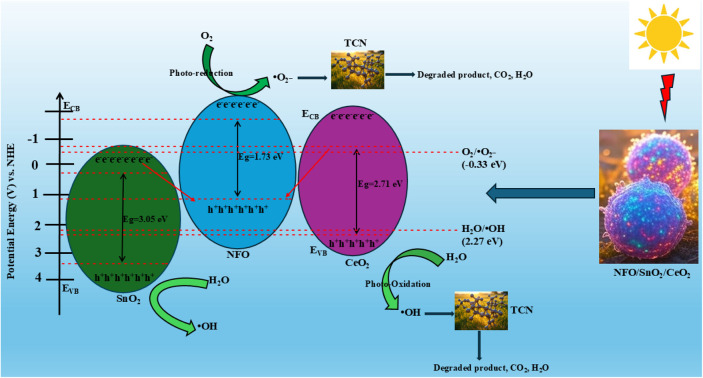

## 1. Introduction

The use of semiconductors as photocatalyst material has over the years attracted much attention as effective materials for removing numerous organic pollutants in water for environmental remediation due to their ability to convert solar energy to chemical energy in environmental remediation applications.^1–3^ Conventional wastewater treatment methods, including biological processes, physical adsorption, chemical oxidation, and membrane filtration, achieve incomplete tetracycline (TCN) removal, as the method usually suffers from high energy demands, secondary pollutant generation, phase transfer without mineralization, and economic barriers that limit scalability.^4^ Advanced oxidation processes like visible-light photocatalysis offer a sustainable alternative by using solar energy to fully degrade TCN into harmless products, but traditional photocatalysts like TiO_2_, ZnO are hindered by wide bandgaps, rapid charge recombination, and low visible-light utilisation, resulting in only 30–60% degradation under sunlight.^5,6^ Over the last decades, many studies have been carried out on developing semiconductor catalysts such as TiO_2_, ZnO, SnO_2_, Fe_2_O_3_, CdS, Ag_3_PO_4_, CdO and ZnS.^7–10^ The application of these semiconductor photocatalysts for the degradation of organic pollutants is limited because their application is only suitable for use under UV light irradiation, which represents only about 5% of the energy in solar radiation, while visible light contributes about 45% of the energy from solar radiation.^11–13^ Therefore, developing new photocatalytic semiconductors with efficient visible-light-driven ability remains challenging. Semiconductor-based ferrite photocatalyst materials have received significant attention in this regard due to their stability, magnetic, and recyclability potential. Nickel ferrite (NiFe_2_O_4_) has proven to be one of the most important spinel ferrites as it exhibits a narrow bandgap of about ∼1.7 eV, low-cost effectiveness, chemical durability, high magneto-crystalline separation, high theoretical specific capacitance (*C*_s_) and exceptional ability for solar light utilisation.^14,15^

However, achieving a high photocatalytic efficiency using pristine NiFe_2_O_4_ for the degradation of organic pollutants in water is limited, possibly due to the fast recombination of photogenerated electron–hole pairs, its small specific surface area, narrow spectral response range, while the availability and cyclability of active sites are decreased when NiFe_2_O_4_ electrodes are used because of their strong propensity to agglomerate, low electrical conductivity, and poor stability during an electrochemical process.^16,17^ In order to overcome these limitations and improve the photocatalytic efficiency and electrode potency of p-type NiFe_2_O_4_, techniques such as doping with noble metals or functionalizing with other semiconductor metal oxides will have to be employed.^18^ reported the construction of a NiFe_2_O_4_/ZnO heterostructure composites where the synergistic effect of nickel ferrite and zinc oxide reduces the probability of recombination of charge carrier and boosts the charge separation resulting in the photocatalytic performance of NiFe_2_O_4_/ZnO to achieve the degradation of 49.2% methyl orange, 44.4% methyl blue and 41.3% crystal violet in 40 min.^9^ reported that the formation of p–n heterojunction between NiFe_2_O_4_ and SQDs enables the direct transfer of photoinduced electrons from NiFe_2_O_4_ to SQDs, which could retard the recombination of electron–hole pairs and enhance the catalytic activity which substantially improved the degradation of rhodamine B upon visible-light treatment within 105 min. The fabrication of the NiFe_2_O_4_/g-C_3_N_4_ heterojunction composite was carried out by Y. Liu *et al.*,^12^ where the synthesised composites achieved 94.5% degradation of tetracycline within 80 min under visible light irradiation. The enhanced photocatalytic activity of the synthesised heterostructure NiFe_2_O_4_/g-C_3_N_4_ composites, when compared to the pristine NiFe_2_O_4_ and g-C_3_N_4_, can be attributed to their increased light utilisation and accelerated photogenerated charge separation during the photocatalysis process.

According to the background and considerations mentioned above, it is evident that combining two semiconductors with suitable energy-matched bands to form heterojunctions is a standard technique to enhance charge separation for extended photocatalysis in a single semiconductor photocatalyst. Many semiconductors such as TiO_2_, SiO_2_/g-C_3_N_4_, LaFeO_3_ and CuO, have been utilised to dope pristine NiFe_2_O_4_ to extend its visible light response.^19–22^ Photocatalyst materials like SnO_2_ and CeO_2_ form a ternary heterojunction with NiFe_2_O_4_ to enhance its photogenerated charge carrier transfer and separation rates. This can be attributed to the SnO_2_ nanostructures' high surface area to volume ratio, broad bandgap, greater binding energy (130 meV), and high carrier mobility of about 250 cm^2^ V^−1^ s^−1^ at ambient temperature, which all contribute to their multifunctionality.^23–25^ While CeO_2_'s narrow band gap, excellent thermal stability, and the Ce^4+^/Ce^3+^ reversible redox pair make it a good option to combine with other semiconductors, where the reversible conversion between Ce^4+^ and Ce^3+^ has been hypothesised to enable effective electron transfer between CeO_2_ and other semiconductors, hence enhancing photocatalytic activity.^25,26^ CeO_2_ also functions as an essential electrocatalytic material for supercapacitors (SCs) due to its quick and reversible conversion of Ce^4+^ to Ce^3+^, which produces oxygen vacancies in the CeO_2_ lattice where the presence of the oxygen vacancies enhances CeO_2_'s electrical conductivity, oxygen availability, and ion and electron transport, all of which boost SC performance.^27,28^

Therefore, in exploring a simple hydrothermal method, the synthesis of novel ternary NiFe_2_O_4_/SnO_2_/CeO_2_ heterostructure composites is presented, where an equimolar amount of SnO_2_ and CeO_2_ was added to NiFe_2_O_4_ for improved photocatalytic activity under visible light irradiation. It is expected that the interaction between the semiconductors; NiFe_2_O_4_, SnO_2_, CeO_2_ can facilitate electron transfer from the reducing catalyst to the oxidising catalyst until Fermi level equilibrium is reached, resulting in the formation of the internal electric field (IEF) and subsequent band bending. The phenomenon of band bending facilitates the interaction between electrons from the oxidation photocatalyst and holes in the reduction photocatalyst. This coulombic interaction enables the separation of charge carriers while maintaining electrons and holes with elevated redox potential.^29^ Furthermore, the obtained ternary nanocomposites were characterised in detail, with the photocatalytic performances of the composite materials declared and the electrochemical potential examined for energy storage. Additionally, a possible photocatalytic mechanism of the ternary nanocomposite was proposed. This work contributes significantly to the field of photocatalysis and wastewater treatment technologies by opening up a new perspective on the link between NiFe_2_O_4_, SnO_2_, and CeO_2_ composites in tackling environmental contamination.

## 2. Materials and methods

### 2.1. Chemicals

The chemicals used in this study were analytical reagent grade and were used without additional purification. Nickel chloride hexahydrate (NiCl_2_·6H_2_O), iron chloride hexahydrate (FeCl_3_·6H_2_O), sodium hydroxide (NaOH), tin chloride (SnCl_4_·5H_2_O), hydrazine monohydrate (N_2_H_4_·H_2_O), cerium(iii) nitrate hexahydrate ((CeNO_3_)_3_·6H_2_O), ethanol, benzoquinone (BQ), isopropyl alcohol (IPA), triethanolamine (TEOA) were supplied by Sigma-Aldrich Co., Ltd South Africa.

### 2.2. Synthesis of NiFe_2_O_4_ (NFO)

For the synthesis of NiFe_2_O_4_, 1 g of iron chloride hexahydrate was dissolved in 100 mL of DI water, followed by stirring for 30 min before the addition of 0.2 g of nickel chloride hexahydrate while stirring for an additional 1 h at room temperature. The pH of the aqueous solution was adjusted to 13 using 4 M NaOH solution to obtain a red-brown solution. The obtained red-brown precipitate was collected, washed several times with DI water and ethanol. The obtained NiFe_2_O_4_ sample were dried in the oven at 70 °C overnight and then calcined in a muffle furnace at 500 °C for 3 h to obtain NiFe_2_O_4_ powder.

### 2.3. Synthesis of SnO_2_

The synthesis of SnO_2_ followed the method previously reported by ref. 24 with slight modification. 1 g of tin chloride was dispersed in 100 mL of DI water and stirred until a homogenous solution was obtained. To the homogeneous solution, 2.5 mL of hydrazine solution was dropwisely added to obtain a white slurry solution. The solution was allowed to stir for 1 h and then transferred into a teflon-lined stainless-steel autoclave, kept at 180 °C in an oven for 24 h. The resulting precipitates of SnO_2_ were obtained *via* centrifugation and subsequently washed several times with deionised water and ethanol. They were then dried overnight in a hot air oven at 80 °C to obtain SnO_2_.

### 2.4. Synthesis of CeO_2_

The hydrothermal method was also utilised in the synthesis of CeO_2_ where 4 g of Ce(NO_3_)_3_·6H_2_O was dissolved in 10 mL of DI water to form solution A while 10 g of NaOH was dissolved in 50 mL of DI water to form solution B. Solutions A and B were mixed together using magnetic stirring for 1 h. The as-obtained mixed white solution was later poured into a Teflon-lined stainless steel autoclave at 100 °C for 24 h. The white precipitate from the hydrothermal treatment was collected by centrifugation and washed several times with DI water and ethanol to remove unreacted reagents. The obtained CeO_2_ was dried in a hot air vacuum oven at a temperature of 70 °C overnight.

### 2.5. Synthesis of NiFe_2_O_4_/SnO_2_/CeO_2_ nanocomposites

Simple hydrothermal techniques were utilised to synthesise novel NiFe_2_O_4_/SnO_2_/CeO_2_ heterostructure composites with equimolar amounts of SnO_2_ and CeO_2_ loading on NiFe_2_O_4_ composites. Typically, 1 g of NiFe_2_O_4_ nanoparticles was dispersed in 100 mL of ethanol and allowed to stir for 1 h at room temperature. An equal amount of SnO_2_ and CeO_2_ was dissolved in a separate beaker containing 20 mL of ethanol. The solutions of SnO_2_ and CeO_2_ were dropwisely added to the solution of NiFe_2_O_4_ under stirring for 1 h before transferring the mixed solutions into a Teflon-lined stainless-steel autoclave and kept at 180 °C for 24 h. The autoclave was allowed to cool to room temperature, and the as-synthesised NiFe_2_O_4_/SnO_2_/CeO_2_ precipitates were obtained by centrifugation and washed several times with DI water and ethanol before drying overnight in an oven at 70 °C. The same hydrothermal techniques were utilised in fabricating NiFe_2_O_4_/SnO_2_ and NiFe_2_O_4_/CeO_2_ nanocomposites.

The SI contains information on the instruments used in characterising the nanocomposites, which includes X-ray diffraction, Fourier Transform Infrared Spectroscopy, field-emission scanning electron microscopy, X-ray photoelectron spectroscopy, UV-vis diffuse reflectance spectrophotometry, photoluminescence spectroscopy, overall organic carbon analysis, photoreactor setup and process for photo degradation.

## 3. Results and discussion section

### 3.1. Physicochemical characterisation

**Fig. 1:**
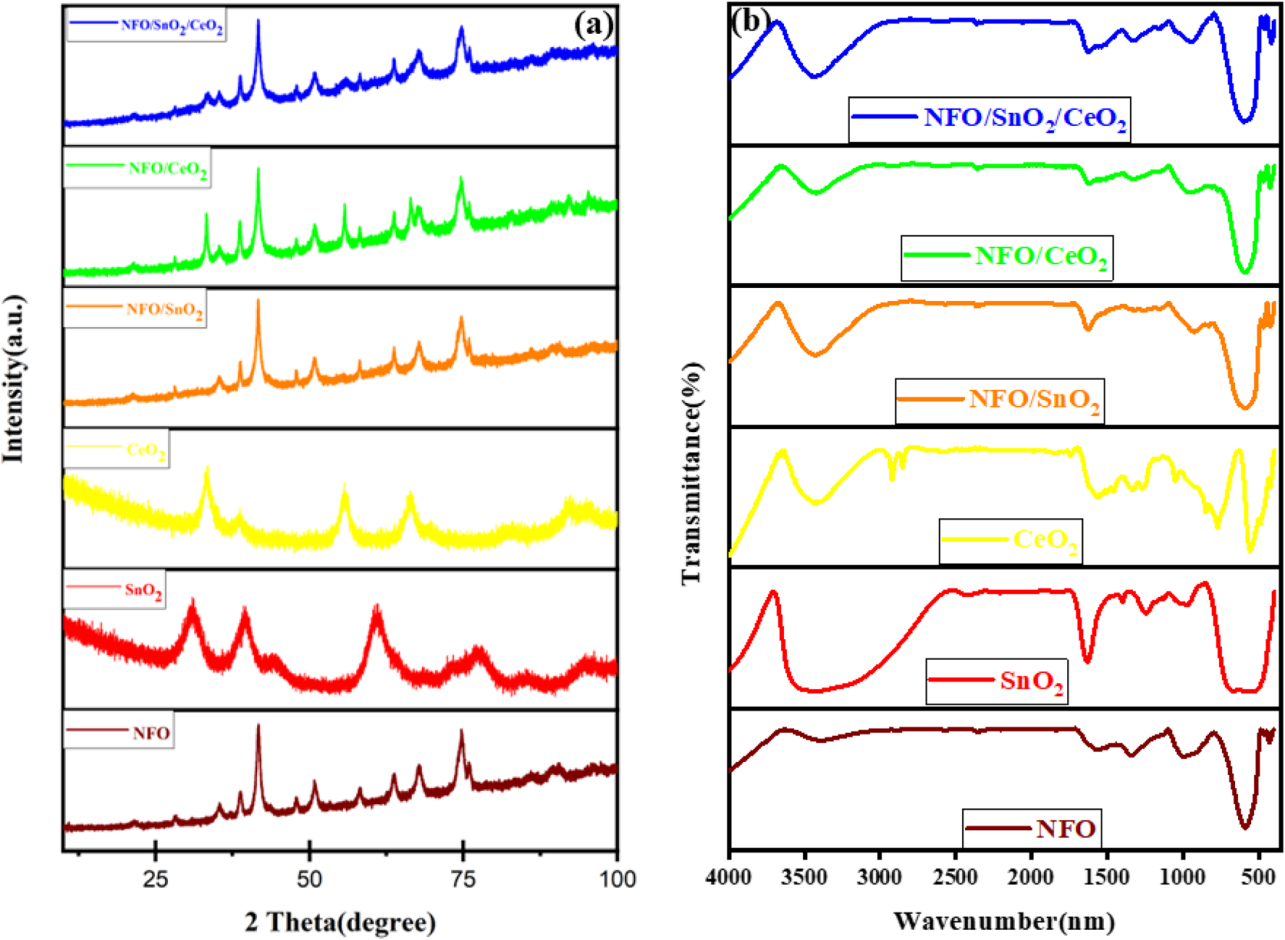
(a) XRD and (b) FTIR patterns of pristine NFO, SnO_2_, CeO_2_ and NFO-based photocatalysts.

Fig. 1 displays the XRD patterns of diffraction peaks of the synthesized materials with NFO showing crystalline peaks at 2*θ* = 35.30, 38.63, 41.65, 47.93, 50.77, 58.21, 63.80, 67.76, 74.80 and 75.95° conforming to the (111), (220), (311), (222), (400), (422), (511), (440), (620) and (533) planes aligned with the intensity peaks of spinel-based cubic (FCC) crystal structure with space group of *Fd*3̄*m* matching with reference ID # 22-1086 (JCPDS file number).^14^ The peaks located at 2*θ* = 33.56, 38.47, 55.66, and 66.48° are attributable to (111), (200), (220), (311) facets of CeO_2_ phase while the XRD pattern of the pristine SnO_2_ composite shows four broad peaks at around 31.05, 39.35, 60.31, and 77.24°, which are indexed to the (110), (101), (211), and (112) planes corresponding to the tetragonal crystal structure of SnO_2_ (JCPDS 01-072-1147).^9^ Additionally, the XRD patterns of NFO/CeO_2_, NFO/SnO_2_, and NFO/CeO_2_/SnO_2_ ternary nanocomposites exhibit similar diffraction spectra corresponding to the diffraction peak of NFO, while the peaks corresponding to CeO_2_ and SnO_2_ are not visibly distinct in the synthesised NFO/CeO_2_, NFO/SnO_2_, and NFO/CeO_2_/SnO_2_ ternary nanocomposites, which can be ascribed to their overlap with the NFO peak or a lower concentration of highly dispersed CeO_2_ and SnO_2_ within the nanocomposites.

The crystallite size of the synthesised photocatalyst was determined using the Scherrer equation, as given below1
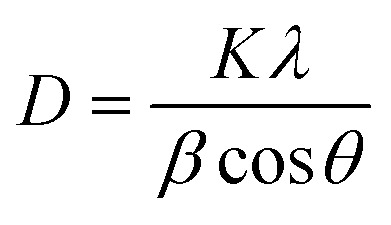
where *D* is the crystallite size in nm, *K* is Scherrer's constant ≈ 0.9, *λ* is the wavelength of the X-ray radiation (Co Kα = 1.789 nm), *β* is the corrected band broadening (full width at half-maximum (FWHM)) of the diffraction peak, and *θ* is the diffraction angle. As shown in Table S.1, the integration of SnO_2_ and CeO_2_ into the NiFe_2_O_4_ lattice resulted in a reduction in the particle/crystallite size of the ternary NFO/SnO_2_/CeO_2_, from 36.49 nm to 10.81 nm which can be attributed to the partial dissolution of the ions of SnO_2_ and CeO_2_ ions into the NFO lattice; this is advantageous for the photocatalytic process, as smaller particle sizes enhance the performance of photocatalysts.^29–31^

FTIR analysis was conducted to confirm the presence of various functional groups in the synthesised nanocomposite materials. As shown in Fig. 1b, the absorption bands at around 430 and 588 cm^−1^ for the FTIR spectrum of NFO are linked to the vibrations of octahedral complexes and intrinsic vibrations of tetrahedral complexes sites of positive ions of NiFe_2_O_4_, as a result of the different values of Fe^3+^ and –O^2−^ distance for octahedral and tetrahedral sites.^32^ The absorption band at 1340 cm^−1^ can be assigned to the anti-symmetric NO stretching vibrations resulting from the nitrate group which is present as a residue in the sample. The absorption bands at 1578 cm^−1^ and 3384 cm^−1^ correspond to the –CH_2_ bending vibration hydroxide and H–O–H bending vibrations of water.^18,33^ For the synthesised composites of CeO_2_, the characteristic absorption bands at around 556 and 772 cm^−1^ are attributed to Ce–O stretching vibration and to the V–O vibration. The absorption peak at 1563 cm^−1^ is characteristic of the physical absorption of the hydroxyl group in CeO_2_, while the bands around 3440 cm^−1^ correspond to the asymmetric stretching of water molecules.^34^ For the composites of SnO_2_, the absorption peaks around 500–600 cm^−1^ are characteristic of the Sn–O bond in the SnO_2_, with the peaks around 1637 and 3459 cm^−1^ attributed to the molecular water bending vibration and hydroxyl groups stretching vibration, respectively.^35^ The absorption peaks for NFO/CeO_2_, NFO/SnO_2_, and NFO/CeO_2_/SnO_2_ ternary nanocomposites consist of absorption spectral characteristics similar to NFO, CeO_2_ and SnO_2_.

**Fig. 2:**
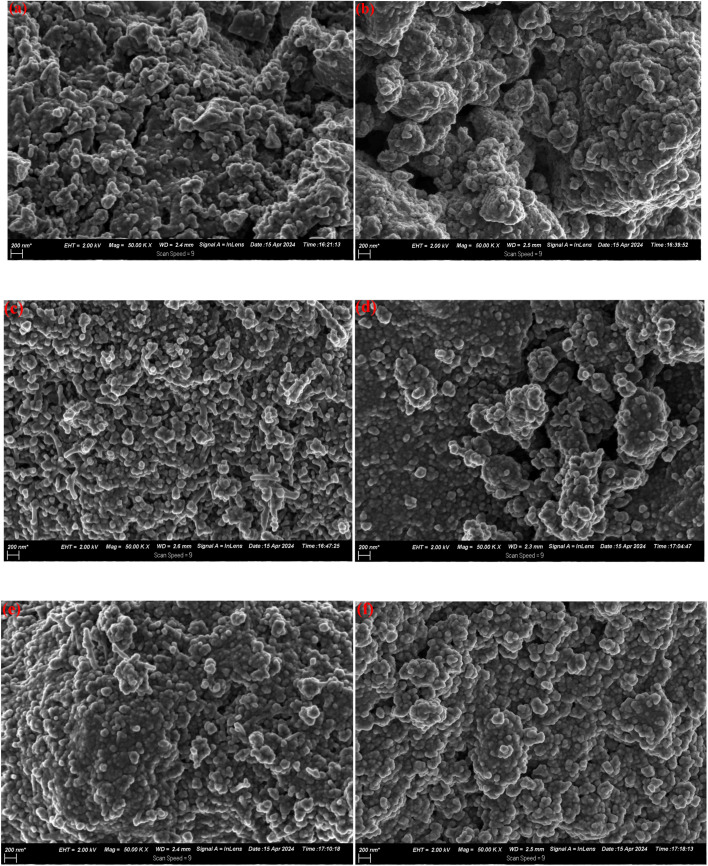
SEM images of the as-prepared samples: (a) NiFe_2_O_4_, (b) SnO_2_, (c) CeO_2_, (d) NiFe_2_O_4_/SnO_2_, (e) NiFe_2_O_4_/CeO_2_ and (f) nanocomposite NiFe_2_O_4_/SnO_2_/CeO_2_.

The morphological features of NiFe_2_O_4_, SnO_2_, CeO_2_ and NiFe_2_O_4_-based photocatalysts were characterised on the basis of the SEM images presented in Fig. 2. For the as-synthesised NiFe_2_O_4_ composites revealed an agglomerate of nanoparticles devoid of even distribution due to their intrinsic properties and high surface energy, which tend to aggregate to minimise their surface energy.^36^ SnO_2_ exhibited spherical-shaped particles with a tightly distributed range of nanoparticle sizes, while the microstructure of CeO_2_ exhibits a rod-like particle state which are closely stacked together. The SEM images of NiFe_2_O_4_/SnO_2_ and NiFe_2_O_4_/CeO_2_ exhibit some agglomeration processes with wrinkle layers of SnO_2_ and CeO_2_ arranged on the surface of NiFe_2_O_4_ as the metallic nanoparticles are easily agglomerated due to hydrophilic hydroxyl saturated residual bonds in different states on their surface. Additionally, SnO_2_ and CeO_2_ show good dispersion on the surface of NiFe_2_O_4_ as they are well coated on the NiFe_2_O_4_ nanoparticles to form an improved agglomerate. The dispersion of SnO_2_ and CeO_2_ on the surface of NiFe_2_O_4_ can greatly increase the specific surface area of the ternary nanocomposites, which provides additional electrochemically active sites during the photodegradation process and helps to improve the electrochemical performance of the electrode material for energy storage, thereby showing the multifunctionality of SnO_2_ and CeO_2_.^37,38^

**Fig. 3:**
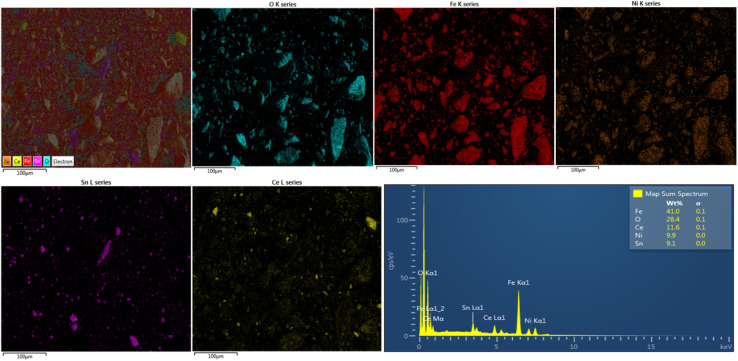
Elemental mapping and EDS spectra of ternary nanocomposite NFO/SnO_2_/CeO_2_.

EDS analyses were done to verify the purity of the nanocomposites and provide insight into the surface morphology and chemical composition of the synthesised ternary NiFe_2_O_4_/SnO_2_/CeO_2_ nanocomposites. The result confirmed the presence of O, Fe, Ni, Sn and Ce elements in the nanocomposite NiFe_2_O_4_/SnO_2_/CeO_2_, while the elemental mapping of Fig. 3 shows the uniform distribution of the five elements in the nanocomposites, which further verifies the successful synthesis of the nanocomposite NiFe_2_O_4_/SnO_2_/CeO_2_ catalyst.

**Fig. 4:**
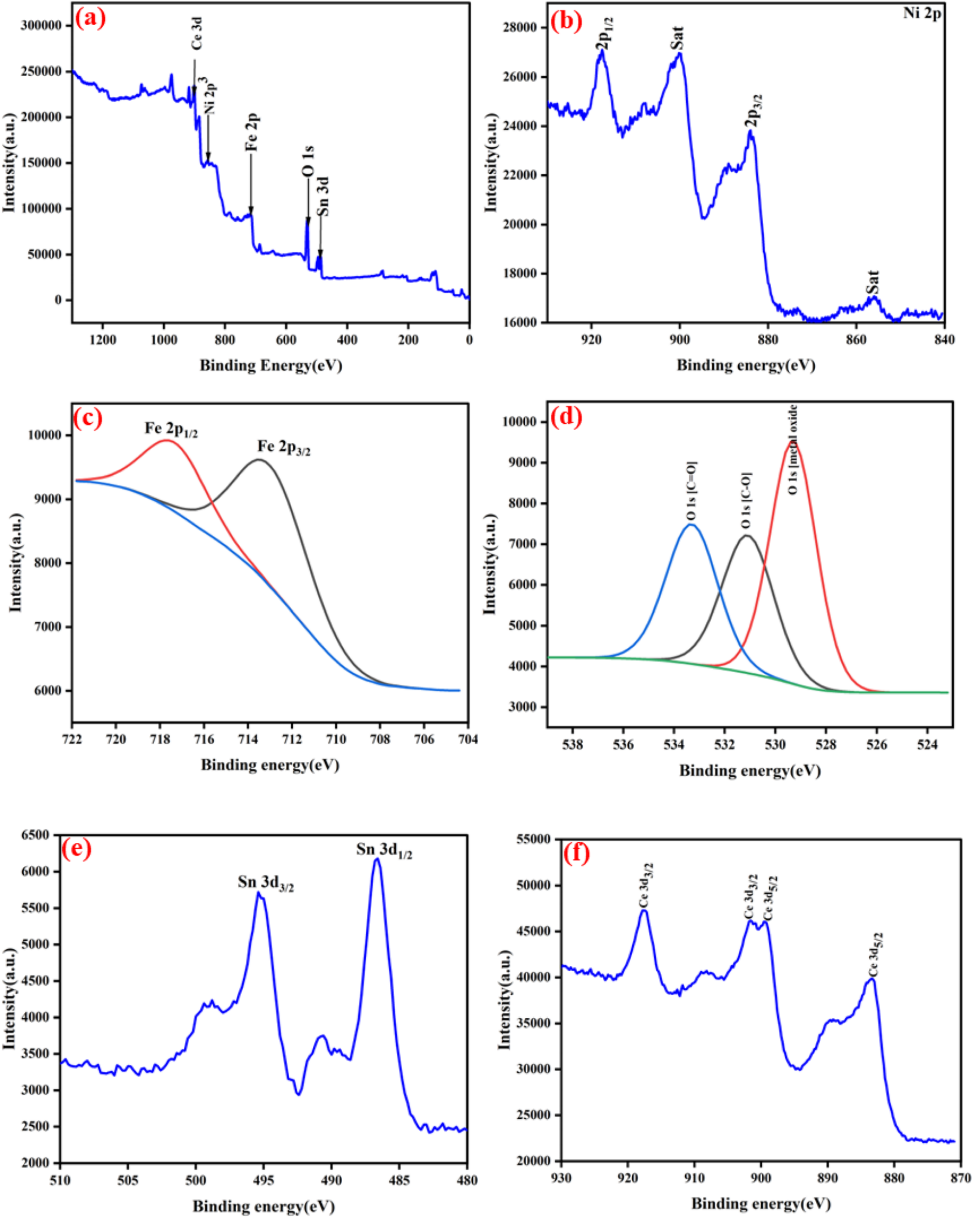
(a) XPS survey scan of NFO/SnO_2_/CeO_2_, (b) Ni 2p, (c) Fe 2p, (d) O 1s, (e) Sn 3d, and (f) Ce 3d.

The physicohemical composition and oxidation state of the synthesised NFO/SnO_2_/CeO_2_ ternary nanocomposite were analysed using X-ray photoelectron spectroscopy (XPS). The XPS ternary nanocomposite of NFO/SnO_2_/CeO_2_ full spectra are illustrated in Fig. 4a, with the presence of the elements Ni, Fe, O, Sn and Ce observed in the survey spectrum of NFO/SnO_2_/CeO_2_ ternary nanocomposite. Fig. 4b depicts the XPS spectrum of Ni 2p element with peaks located at 856.2 eV, 884.2 eV, 900.2 eV and 917.6 eV with the peaks at 884.2 eV and 917.6 eV corresponding to Ni 2p_3/2_ and Ni 2p_1/2_ which is typical for the presence Ni^2+^ and Ni^3+^ while the peaks at 900.2 eV and 856.2 eV are satellite peaks in the synthesized nanocomposites.^14,39^ For the Fe 2p spectrum, the characteristic peaks at binding energies 713.4 eV and 717.8 eV correspond to the Fe 2p_3/2_ and Fe 2p_1/2_, which confirms the presence of Fe^2+^ and Fe^3+^ oxidation states. The O 1s spectrum (Fig. 4d) shows three distinct peaks at 529.4 eV, 531.2 eV and 533.4 eV attributable to the combination of lattice oxygen O^2−^ with M-bonding, while the peak at 533.4 eV is attributed to the presence of adsorbed oxygen in the synthesised nanocomposites. During the photocatalytic process, the adsorbed oxygen can generate free radical active species through the absorption of light-generated electrons.^40,41^Fig. 4e shows the spectrum of the Sn 3d state, which exhibits two distinctive asymmetric peaks at higher binding energies of 486.6 eV and 495.5 eV, corresponding to the Sn 3d_5/2_ and Sn 3d_3/2_ states, respectively. The spin–orbit split of approximately 8.90 eV caused by the binding energy is suggestive that the Sn element is present in the synthesised ternary nanocomposites as Sn^4+^.^42,43^Fig. 4f displays the deconvoluted spectrum of Ce 3d of distinct peaks with binding energies at around 883.4 eV and 899.4 eV, which are attributed to the Ce 3d_5/2_, while peaks with binding energies at around 901.8 eV and 917.6 eV are attributed to Ce 3d_3/2_ states, which indicate the valence state of Ce^3+^.^34,44^

**Fig. 5:**
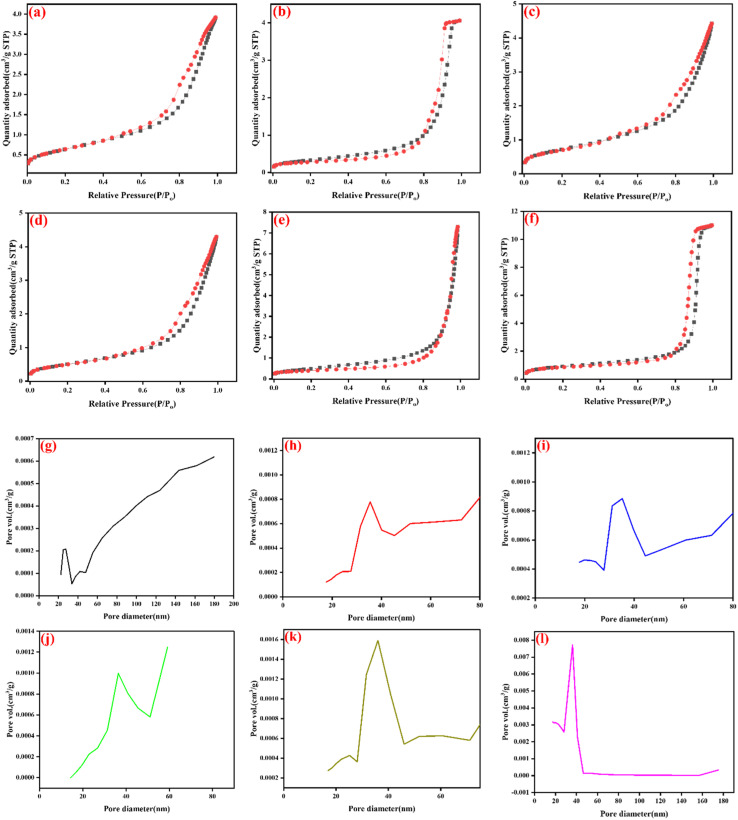
N_2_ adsorption–desorption isotherms (a) NFO, (b) SnO_2_, (c) CeO_2_, (d) NFO/SnO_2_, (e) NFO/CeO_2_, (f) NFO/SnO_2_/CeO_2,_ with its pore size distribution plot of (g) NFO, (h) SnO_2_, (i) CeO_2_, (j) NFO/SnO_2_, (k) NFO/CeO_2_, (l) NFO/SnO_2_/CeO_2_ for the synthesised materials.

The surface physicochemical properties of the synthesised nanocomposite catalyst were investigated using the N_2_-BET (Nitrogen adsorption Brunauer–Emmett–Teller) to examine their specific surface area, pore volume and pore diameter. As depicted in Fig. 5, all the synthesised composite materials display type-IV isotherms with H3 hysteresis loops based on IUPAC classification, which indicates that all the composite materials have standard mesoporous structures.^45,46^ The specific surface area of the synthesised NFO, SnO_2_, CeO_2_, NFO/SnO_2_, NFO/CeO_2_, and the nanocomposite NFO/SnO_2_/CeO_2_ were 26.82, 11.77, 41.58, 38.71, 51.93 and 72.99 m^2^ g^−1^, respectively. The pore volume, estimated using the BJH model, were 0.00022, 0.0078, 0.00085, 0.00090, 0.0016 and 0.0077 cm^3^ g^−1^, respectively, for NFO, SnO_2_, CeO_2_, NFO/SnO_2_, NFO/CeO_2_ and NFO/SnO_2_/CeO_2_. The pore diameter, estimated using the BJH model, was 27.56, 35.46, 35.20, 36.45, 36.27 and 38.99 nm for pore NFO, SnO_2_, CeO_2_, NFO/SnO_2_, NFO/CeO_2_ and NFO/SnO_2_/CeO_2_, Table S.1. shows these values. Therefore, it is obvious that the BET specific surface area, pore volume and diameter of the ternary nanocomposite NFO/SnO_2_/CeO_2_ material/catalyst is higher than the values for NFO, SnO_2_, CeO_2_, NFO/SnO_2_ and NFO/CeO_2_, attributable to the addition of CeO_2_ and SnO_2_ as co-catalysts. These co-catalysts, with high and low molecular weights, contributed to the formation of heterojunctions within the nanocomposites, creating an interface where electrons and holes can transfer more efficiently. This is crucial for photocatalysis, as it prevents the recombination of electron–hole pairs, which would otherwise limit their ability to participate in redox reactions. Hence, the improvement in the ternary's specific surface area and porosity will be advantageous during the photocatalytic process, generating more catalytically active sites for the absorption of pollutants and thereby hastening their removal under visible light illumination.^47^

### 3.2. Optical and electrochemical performance

**Fig. 6:**
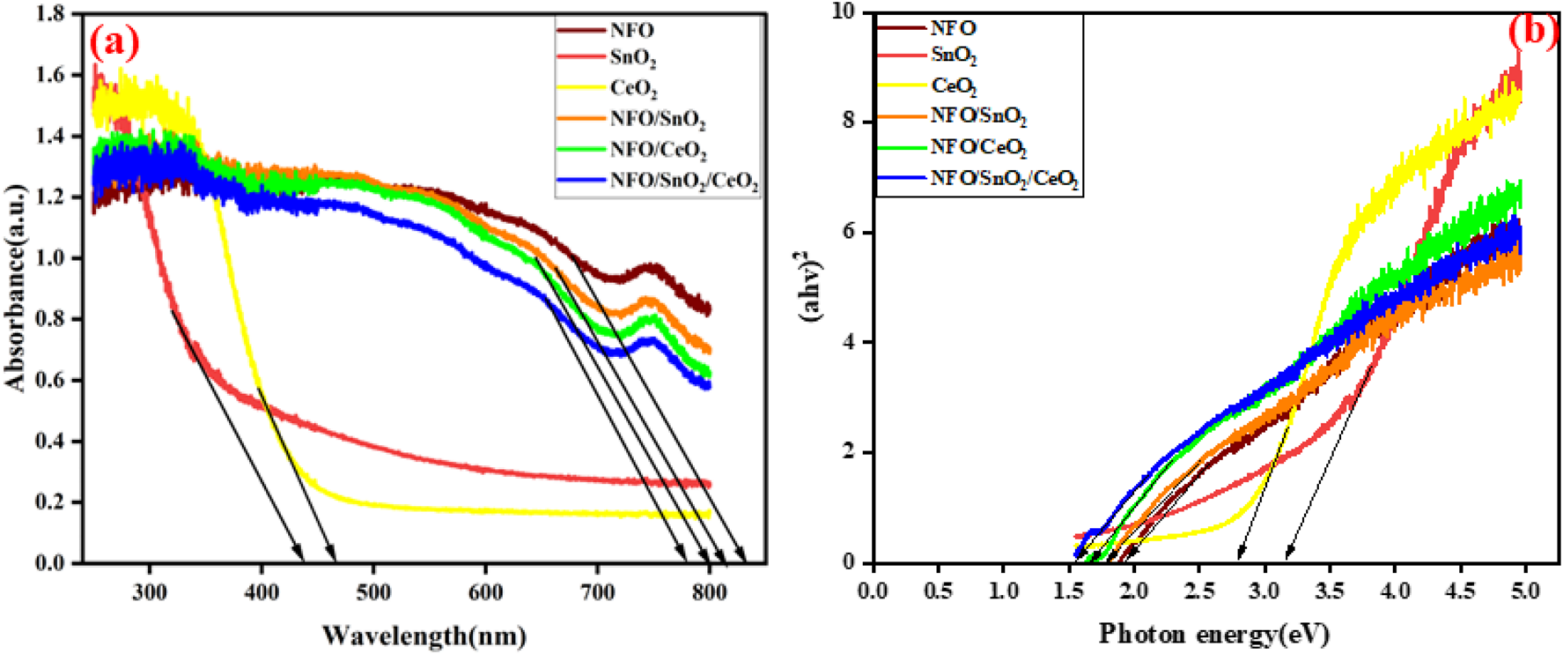
(a) UV-vis diffuse reflectance spectroscopy spectra and (b) Tauc plots of the synthesised materials.

The UV-vis diffuse reflectance spectra of the synthesised materials were obtained to evaluate their optical properties. The results as represented in Fig. 6, the NFO material shows an absorption edge in the visible region at about 780 nm while SnO_2_ and CeO_2_ display absorption edges appearing at round 436 and 466 nm, respectively. The absorption edge of the ternary NFO/SnO_2_/CeO_2_ nanocomposites is significantly red-shifted at about 832 nm due to the incorporation of SnO_2_ and CeO_2_ as co-catalyst to enhance the optical absorption capacity of NFO within the visible light region, resulting in improved electron–hole pair generation and increased generation of active species radicals, thereby promoting its photocatalytic potential.

Using the Tauc equation as given below, the energy band gap of NFO, SnO_2_, CeO_2_, NFO/SnO_2_, NFO/CeO_2_, and NFO/SnO_2_/CeO_2_ nanocomposite materials were calculated from the spectra to be 1.72, 3.05, 2.71, 1.65 and 1.53 eV, respectively, as shown in the expanded plot in Fig. S.1.

2 *αhν* = *A*(*νh* − *E*_g_)^*n*/2^

The results show that the integration of SnO_2_ and CeO_2_ as co-catalysts decreases the band gap of NFO, thereby expanding its range of visible light absorption, which is beneficial for generating more electron–hole pairs during the photocatalytic process.

**Fig. 7:**
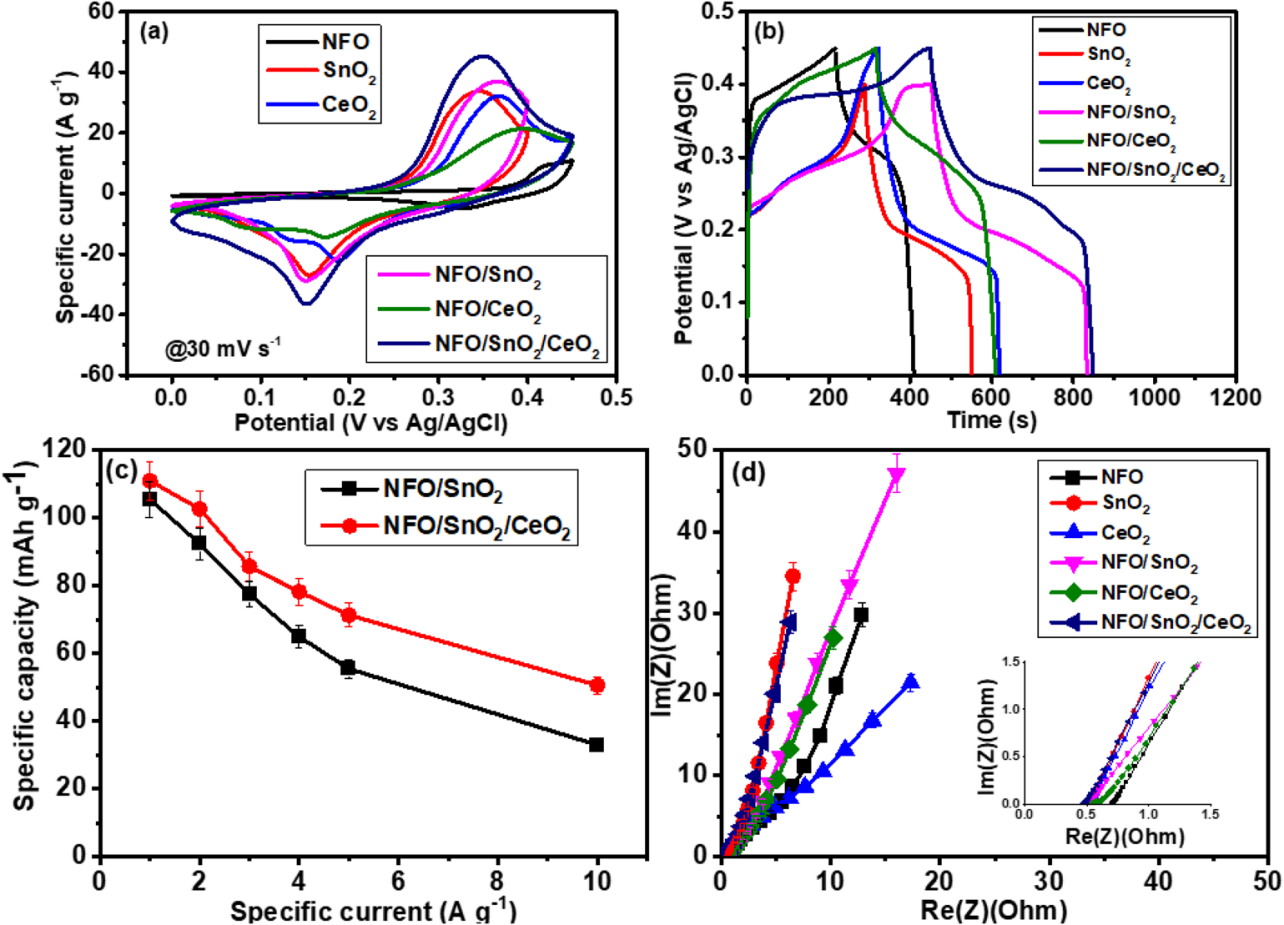
Comparison of (a) CV at 30 mV s^−1^, (b) GCD at 1 A g^−1^, (c) specific capacity *versus* specific current of NFO/SnO_2_ and NFO/SnO_2_/CeO_2_, (d) Nyquist plot (with an inset at high frequency).

Energy research is currently focused on finding affordable electroactive materials with high specific capacity. Metal oxides (MOs) are preferred for energy storage applications, such as electrode materials in electrochemical capacitors, because of their high capacity.^48^ Therefore, the synthesized materials were further evaluated as electrode materials for supercapacitor applications. Cyclic voltammetry (CV), galvanostatic charge–discharge (GCD), and electrochemical impedance spectroscopy (EIS) were investigated over a frequency range of 100 kHz to 10 mHz. As shown in Fig. 7, the electrochemical performance of the synthesised samples, NFO, SnO_2_, CeO_2_ and their nanocomposites were tested using 6.0 M KOH electrolyte. Fig. 7a exhibits a comparison of CV curves for all the synthesized materials, tested at a scan rate of 30 mV s^−1^. It displays similar profiles of sharp redox peaks in all the CV curves, owing to pseudo-capacitive behaviour in the system, which is demonstrative of the reversible redox (charge transfer) reactions occurring on the surface of the electrode materials.^48,49^ The observed shape within all the synthesised samples is attributed to a characteristic profile of faradaic behaviour, while the GCD outcome showed nonlinear curves with well-defined charge and discharge plateaus, designating the typical faradaic behaviour of the electrode materials as depicted in Fig. 7b.^50,51^ It is important to notice that all the tests were done in a wide potential window ranging from 0.0 to 0.45 V *vs.* Ag/AgCl, except SnO_2_ and NFO/SnO_2_ composite tested in a less wide potential window (0.0 to 0.40 V *vs.* Ag/AgCl) due to electrolyte decomposition. However, all the samples display oxidation and reduction peaks. The maximum specific capacity (*C*_sp_) recorded for each sample was measured from the GCD curves at a current density value of 1 A g^−1^, using eqn (3) as given below.^52,53^3
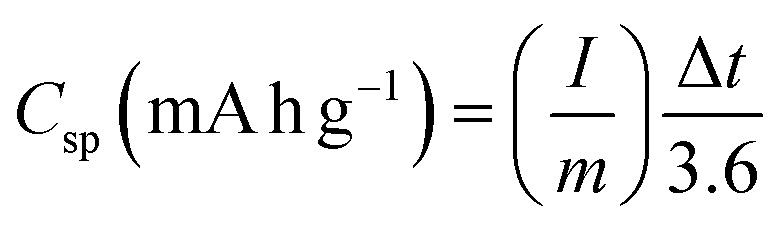
where *I* is the current (mA); *m*, the mass of the active material (g); and Δ*t*, the discharge time (s).

NFO electrode material recorded a specific capacity of 53.5 mA h g^−1^, which is promising when compared to some recently reported results, which show an inferior discharge time.^51,54^ Oxidation and reduction are represented by the pair of peaks observed in all samples in CV and GCD, indicating that the faradaic reaction is occurring during the procedure. Consequently, the following equation can be used to describe the electrochemical reaction mechanism in the NFO electrode material, as shown below.

4 NiFe_2_O_4_ + H_2_O + OH^−^ ↔ NiOOH + 2FeOOH + e^−^

SnO_2_ electrode material displayed good electrochemical properties with a specific capacity of 73.4 mA h g^−1^, superior to that of NFO. This can be attributed to the low electrical conductivity of NFO compared to SnO_2,_ while the charge storage mechanism of SnO_2_ with the aqueous electrolyte at the electrode/electrolyte interface can be detailed as shown in eqn (5)–(7) below:

5 SnO_2_ + *x*K^+^ + *x*OH^−^ → K_*x*_SnO_2_

6 SnO_2_ + H_2_O + e^−^ ↔ SnOOH + OH^−^

7 SnOOH + e^−^ ↔ SnO + OH^−^

Compared to recent similar studies, our synthesised materials displayed better performance as an electrode for supercapacitor applications because of their longer discharge time. Recently studies like,^50^ the authors reported a Ni-doped SnO_2_ nanocomposite, and they reported a highest specific capacity of 61.01 mA h g^−1^ when compared to 69.3 mA h g^−1^, obtained using the same electrolyte of 6 M KOH at a specific current of 2 A g^−1^. In the same vein,^55^ reported that ternary nanocomposite electrode, 3DG–SnO_2_–TiO_2_, delivered a maximum specific capacity of 232.7 C g^−1^ at 1 A g^−1^ which is approximately equal to 64.6 mA h g^−1^. Additionally, our CeO_2_ displayed a high specific capacity of 82.8 mA h g^−1^, which is also much better than some similar studies in literature. For instance, CeO_2_/Mg–NH_4_F composite prepared and tested by ref. 56 displayed a maximum specific capacity of 172 C g^−1^ (approximately 47.7 mA h g^−1^) while ref. 57 reported a specific capacitance of 91.75 F g^−1^ for their CeO_2_ nanoparticle. These materials still exhibit lower performance compared to the performance obtained in the present study. As demonstrated previously, all samples exhibit faradaic behaviour; herein, charge storage can be explained based on the reversible electrochemical adsorption of electrolyte cations onto the electrode surface. This can be expressed *via* the following equations:CeO_2_ + K^+^ + e^−^ ↔ CeO_2_K^+^CeO_2_ + H_2_O + e^−^ → Ce^3+^OOH + 2OH^−^

8 Ce^3+^OOH − e^−^ + 2OH^−^ → CeO_2_ + H_2_O

Potassium ions, K^+^ from the KOH electrolyte are adsorbed onto the surface of the CeO_2_ electrode, while during discharge, Ce^4+^ in the CeO_2_ lattice interacts with K^+^ ions and gains electrons. This leads to the reduction of Ce^4+^ to Ce^(4−*x*)+^. Whereas, during the charging process, Ce^(4−*x*)+^ loses an electron and changes toward Ce^4+^.

Thereafter, the prepared binary NFO/CeO_2_ and NFO/SnO_2_, as well as the ternary NFO/SnO_2_/CeO_2_ nanocomposite materials, revealed greater electrochemical performance compared to the pristine materials, with specific capacity values of 81.4, 105.5, and 106.7 mA h g^−1^, respectively. These favourable electrochemical results can be attributed to a significant synergistic effect between the different components, which enhances the charge storage capacity in the composite materials. The existence of multiple redox properties contributes to producing high specific capacity during the reaction pathway. Furthermore, the NFO electrode exhibits low electrical conductivity, strong agglomeration tendencies, and poor stability during electrochemical reactions, which reduces the availability and cyclability of active sites.^58^ Therefore, developing composite materials is an efficient strategy to overcome these limitations and increase the specific capacity. Fig. 7c displays the specific capacity as a function of the specific currents, ranging from 1 to 10 A g^−1^, for the best composite materials (NFO/SnO_2_ and NFO/SnO_2_/CeO_2_). We can see that the ternary composite recorded a superior specific capacity with a good rate capability.

Remarkably, the area integral of the CV plots for this electrode is higher compared to those for NFO/SnO_2_ and the other electrodes, indicating that the presence of multiple redox properties significantly enhances the electrochemical reaction activity and leads to better SC performance. Additionally, it exhibits a good rate capability, from low to high specific current, compared to the binary composite. An EIS Nyquist plot study was performed and as displayed in Fig. 7d two regions can be observed: the low-frequency region and the high-frequency region, where all the synthesized materials show a very low equivalent series resistance (*R*_s_) at the high-frequency region. The calculated values are as follows: 0.70, 0.50, 0.51, 0.47, 0.52 and 0.54 Ω for NFO_2_, SnO_2_, CeO_2_, NFO/SnO_2_, NFO_2_/CeO_2_ and NFO_2_/SnO_2_/CeO_2_, respectively. These values were obtained from the intercept point between the curves and the *x*-axis, and as seen in the inset to Fig. 7d, the absence of visible semi-circles demonstrates a low resistance charge transfer (*R*_ct_), leading to good ionic conductivity. At the low frequency portion, approximate linear components with short diffusion length are noted, suggesting rapid kinetics diffusion progression with low resistance.^50,59^ Thereby, NFO/SnO_2_ binary and NFO/SnO_2_/CeO_2_ ternary composites recorded better electrochemical performance. However, NFO/SnO_2_/CeO_2_ outperforms NFO/SnO_2_, which can be attributed to greater synergistic effects and superior redox properties, resulting in a much wider stable working potential window that can help produce a high specific capacity. Therefore, the ternary composite exhibited a higher specific capacity (110.7 mA h g^−1^), a smaller *R*_s_ (0.46 Ω), and maintained good rate capability, which allows us to investigate this sample further.

**Fig. 8:**
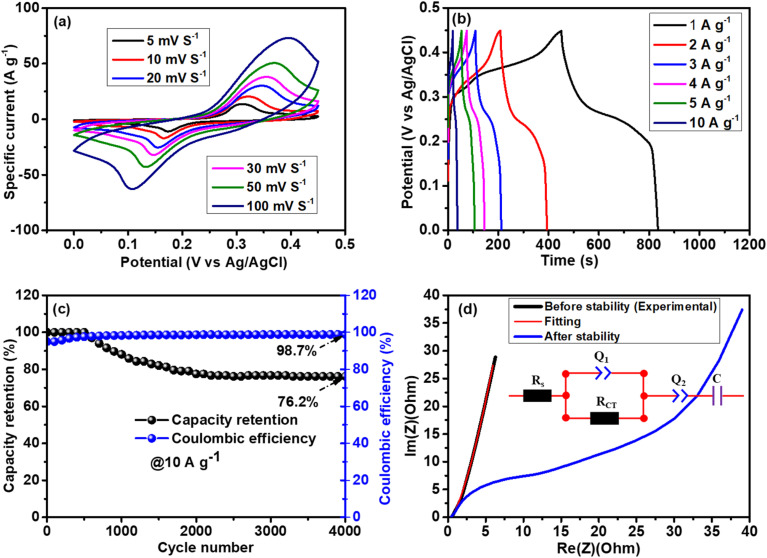
(a) CV curves at different scan rates, (b) GCD plots at different current densities, (c) coulombic efficiency and capacity retention as function of cycle number, and (d) EIS Nyquist plot: experimental (before and after stability) and fitting with the inset of equivalent circuit diagram used to fit the EIS for NFO/SnO_2_/CeO_2_.

Fig. 8a illustrates the cyclic voltammograms (CV) of NFO/SnO_2_/CeO_2_ obtained at different scan rates ranging from 5 to 100 mV s^−1^ within a 0–0.45 V *vs.* Ag/AgCl working potential window. From low to high scan rates, the shape of the CV remains unchanged, and a clear redox peak is still visible even at 100 mV s^−1^, demonstrating fast Faraday-type reactions with good reversibility. As the scan rate is raised from 5 to 100 mV s^−1^, the redox peaks move to positive and negative directions; this phenomenon is linked to the high internal diffusive drag in this system.^60^ To further assess the potential of this electrode material, the GCD was implemented at various specific currents ranging from 1 to 10 A g^−1^. Fig. 8b illustrates the corresponding GCD plots, where it is observed that the charge/discharge time decreases with an increase in specific current. This is because at high current densities, electrolyte ions and charges do not have sufficient time to enter the inner surface of the electrode materials; therefore, the only outer electrode surface undergoes electrochemical reactions. It can be noted that from low to high density, the electrode maintains the same shape profile with the presence of the redox peaks. Fig. 8c displays the electrode's cyclic performance *versus* the cycle number of the ternary composite NFO_2_/SnO_2_/CeO_2,_ where the electrode showed an excellent coulombic efficiency of 98.7% and maintained a good specific capacity of 74.5% up to 4000 galvanostatic charge/discharge at 10 A g^−1^. Overall, the prepared materials demonstrated excellent electrochemical performance and are thus suitable for energy storage applications.

Fig. 8d shows the Nyquist plot of NFO/SnO_2_/CeO_2_ before and after cycling, along with the fit (red line) using the equivalent circuit diagram shown in the inset. The plots show the same *R*_s_ of 0.46 Ω after the electrode was subjected to the cycling stability test. *R*_s_ represents the overall resistance of the SC system, including the intrinsic resistance of the active material, the contact resistance at the electrode/current collector interface, and the electrolyte ionic resistance.^61^ However, there is an increase in diffusion length with a deviation from the *y*-axis, which may be related to a decrease in ionic conductivity due to a reduction of ions in the electrolyte after repeated charging and discharging. A degradation of the electrode surface after cycling can also cause an increase and decrease in diffusion length and conductivity, respectively.^53,62^ At high and mid-frequencies, the equivalent circuit diagram (Fig. 8b) shows a *R*_s_ in series with RCT, which is in parallel to the real capacitance, Q1, representing the pseudocapacitance of the redox sites, respectively. At low frequencies, vertical line-like behaviour is observed close to the imaginary axis, *Q*2, in series with a nearly ideal capacitance (*C*). *Q*2 can also be linked to the diffusion related to the non-ideal capacitance.

### 3.3. Photocatalytic evaluation

The visible light performance of the synthesised catalysts was examined by degradation of tetracycline (TCN) in water, as plotted in Fig. 8. Each catalyst exhibits some degree of adsorption capacity for tetracycline within the first 30 min, achieving adsorption levels of 17.55, 3.82, 7.23, 18.57, 19.53, and 30.05%, which can be attributed to the ionisation properties of TCN and the surface charge characteristics of the catalyst.^63^ Throughout the photolysis procedure, the TCN concentration remained essentially constant, with only 8.66% degradation observed, indicating that self-degradation was insignificant. The synthesised NFO achieved 57.73% degradation, while SnO_2_ and CeO_2_ exhibited low photocatalytic degradation efficiencies of 19.57% and 45.42% for TCN degradation under visible-light irradiation for 1 h. Significantly, the photocatalytic efficiency of NFO/SnO_2_ and NFO/CeO_2_ was markedly enhanced with the incorporation of SnO_2_ and CeO_2_ into the NFO matrix, which showed notable photocatalytic performance of 68.99 and 73.12% for the degradation of TCN. After irradiation for 1 h under visible light, the degradation rate of TCN reached 97.92% using the NFO/SnO_2_/CeO_2_ nanocomposites. The improvement in the degradation efficiency of the ternary NFO/SnO_2_/CeO_2_ nanocomposites can be attributed to a significant synergistic effect at the core/shell interface of the NFO, SnO_2_, and CeO_2_ photocatalysts linked to the formation of a heterostructure between them, which promotes the effective transfer of photogenerated carriers.

**Fig. 9:**
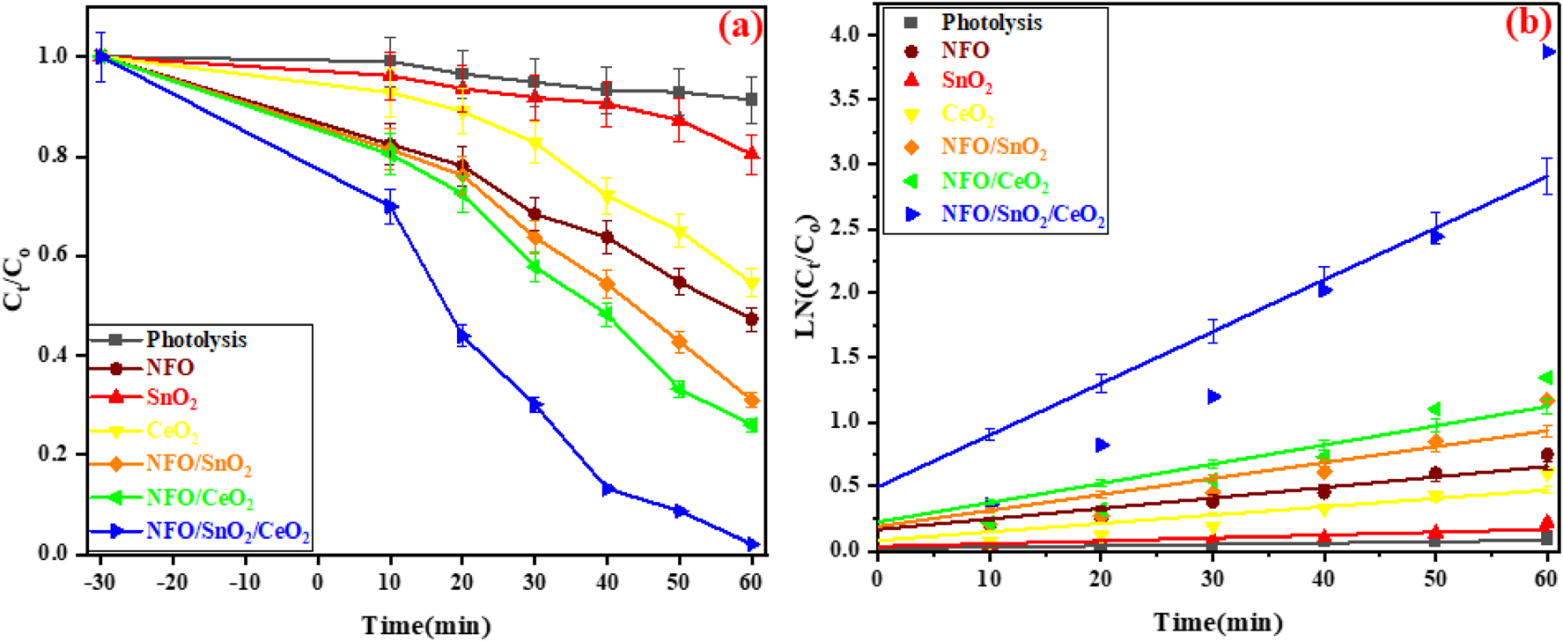
(a) Photocatalytic degradation of TCN and (b) pseudo-first-order kinetics of TCN degradation over the synthesised composite materials.

As shown in Fig. 9b, the corresponding kinetic linear fitting curves of the synthesised catalyst are consistent with the basic characteristics of first-order kinetics. The degradation rate constant of NFO/SnO_2_/CeO_2_ can reach 0.04018 min^−1^, which is 5 times larger than that of NFO (0.00818 min^−1^), 19 times larger than that of SnO_2_ (0.00215 min^−1^), 6 times larger than that of CeO_2_ (0.00649 min^−1^), 3.2 times larger than that of NFO/SnO_2_ (0.00124 min^−1^) and 2.7 times larger than that of NFO/CeO_2_ (0.01497 min^−1^).

**Fig. 10:**
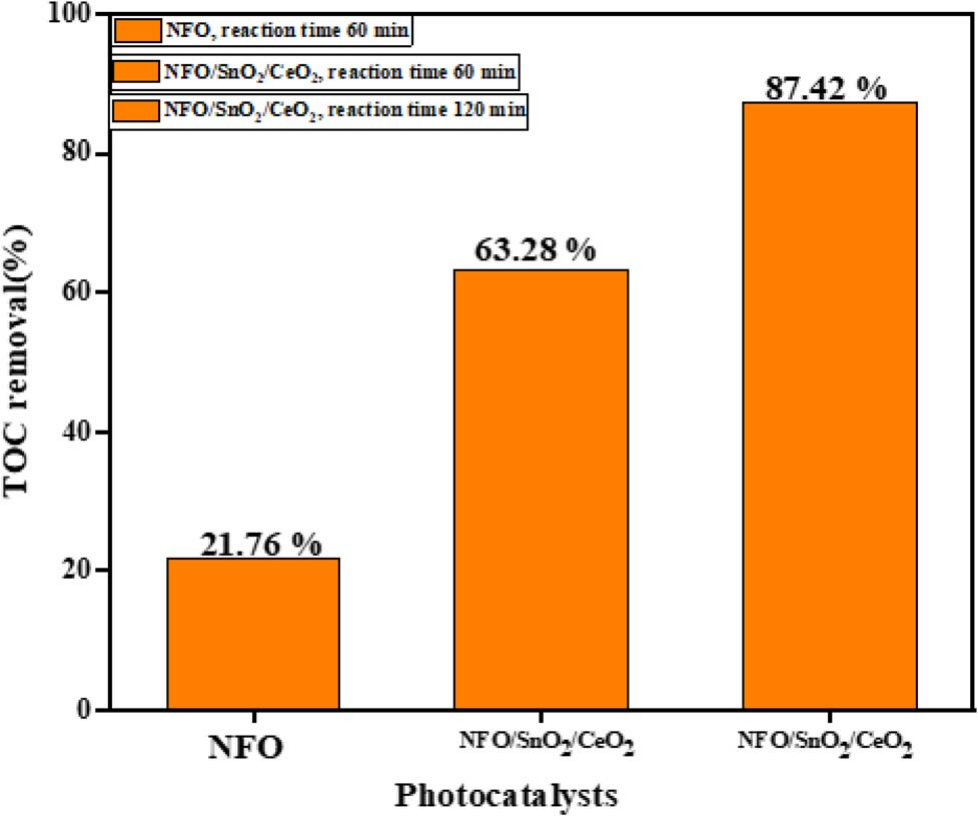
TOC removal by pure NFO, and ternary nanocomposite NFO/SnO_2_/CeO_2_ catalyst.

We employed TOC analysis to further ascertain the degree of TCN mineralisation during photocatalysis in order to confirm the mineralisation impact during the catalytic degradation of TCN, as illustrated in Fig. 9. The NFO and NFO/SnO_2_/CeO_2_ heterojunction photocatalysts' degradation mineralisation efficiency, as determined by analysis and calculation, were 23.64% and 63.28%, respectively. These rates were somewhat lower than the degradation rates during the photocatalytic stage. However, there were comparable patterns in the photocatalytic degradation curve and the TOC removal rate. This is due to the fact that there are still trace amounts of intermediates in the solution that are not entirely transformed into inorganic substances. However, the TOC removal over the ternary nanocomposites increased to 87.42% when the illumination time was extended to 120 min, confirming a promising application potential for wastewater treatment (Fig. 10).

Furthermore, the degradation rate of the ternary NFO/SnO_2_/CeO_2_ nanocomposite was compared with other documented photocatalysts for the degradation of TCN. As shown in Table S.2, the photocatalytic performance of the ternary nanocomposites for TCN degradation is competitive with the reported photocatalysts. It should be noted that a direct comparison is difficult because of the difference in photocatalytic reaction conditions like pollutant concentration, photocatalyst dosage, reaction time and energy consumed *etc.* However, the ternary nanocomposite NFO/SnO_2_/CeO_2_ catalyst presented in this study displayed excellent visible-light-driven photocatalytic activity for TCN degradation. Therefore, the results demonstrate that the nanocomposite NFO/SnO_2_/CeO_2_ catalyst is a promising photocatalyst for the removal of antibiotic pollutants.

**Fig. 11:**
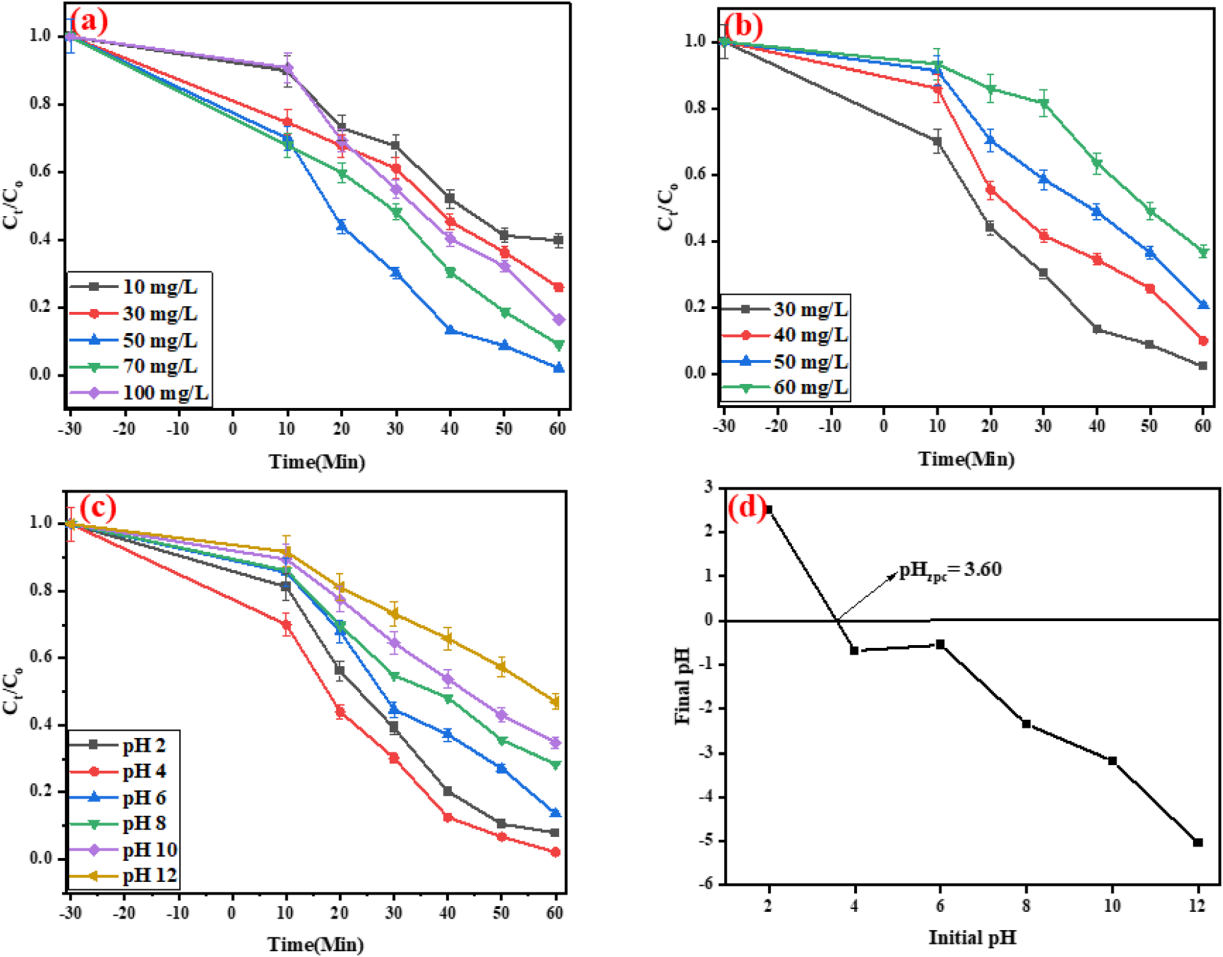
(a) Effect of dosage plot, (b) effect of TCN concentration, (c) effect of pH and (d) point of zero charge (pH_zpc_) using the ternary nanocomposite NFO/SnO_2_/CeO_2_ catalyst.

The degradation performance of the catalyst is significantly influenced by experimental conditions like catalyst dosage and pH of the medium. To obtain the best performance of NFO/SnO_2_/CeO_2_ for the photodegradation of TCN, controlled experiments were carried out to optimise these parameters towards practical applications. For this investigation, the removal effectiveness of TCN increased from 60.15% to 97.92% when the amount of the ternary nanocomposite NFO/SnO_2_/CeO_2_ catalyst was increased from 10 to 50 mg L^−1^, as shown in Fig. 11a. However, the degradation rate was reduced to 90.98% and 83.57%, respectively, when the catalyst dose was increased to 70 and 100 mg L^−1^. This observation could be attributed to the excess catalyst blocking photon absorption, as reported elsewhere.^64^

The degradation efficiency of TCN by the NFO/SnO_2_/CeO_2_ nanocomposites was evaluated using a series of TCN concentrations (30, 40, 50, and 60 mg L^−1^). As shown in Fig. 11b, efficiency gradually declined with increasing TCN concentration above 30 mg L^−1^, decreasing from 97.95% to 63.22%. The decrease in the degradation efficiency of the ternary NFO/SnO_2_/CeO_2_ nanocomposites at higher TCN concentrations can be attributed to two main factors. First, the increased concentration of TCN leads to greater absorption of incident light by the solution itself and by the surfaces of the nanocomposites, which reduces the effective light penetration necessary to activate the photocatalyst. Second, higher concentrations of TCN result in the formation of more intermediate degradation products, which compete with the parent pollutant for active adsorption sites on the composite surface. This competitive adsorption between TCN and its intermediates limits the availability of reactive sites for further degradation, ultimately reducing photocatalytic performance. These factors collectively contribute to the observed decline in efficiency at elevated pollutant concentrations.

There are two primary reasons for the decreased degrading efficiency of the ternary NFO/SnO_2_/CeO_2_ nanocomposites at increasing TCN concentrations. First, the solution and the surfaces of the nanocomposites absorb more incident light due to the higher TCN concentration, which lowers the effective light penetration required to activate the photocatalyst. Second, additional intermediate degradation products are produced at higher TCN concentrations, and these compounds compete with the parent pollutant for active adsorption sites on the composite surface. The availability of reactive sites for further degradation is limited by the competing adsorption between TCN and its intermediates, which ultimately reduces photocatalytic performance. All of these elements work together to cause the efficiency drop observed at high pollution concentrations.^65,66^

As shown in Fig. 11c, TCN degradation efficiency at different pH values: 2, 4, 6, 8, 10 and 12 was investigated to assess the effect of pH on the degradation performance of the catalyst. At pH 4, the TCN degradation rate of the ternary nanocomposite NFO/SnO_2_/CeO_2_ significantly increased to 97.92% while at pH 12, the catalyst displayed the lowest TCN degradation rate, 56.06%. At the pH of 6, 8, and 10, the TCN degradation rate was 86.39%, 71.58%, and 65.24%, respectively. This can be attributed to the morphology of the TCN and the charge distribution on the surface of the nanocomposite, as indicated by the value of the point of zero charge at 3.60, resulting in the generation of active intermediates that facilitate TCN degradation in an acidic medium.^67,68^ TCN being an amphoteric molecules with p*K*_a_ values of 2.8–3.4, 7.2–7.8, and 9.1–9.7, comprises acidic phenolic hydroxyl, enol hydroxyl, and basic dimethylamine groups hence the van der Waals force interactions between the TCN and the nanocomposite's surface enhances the adsorption characteristics when the pH of the solution becomes closer to this value, which promotes the TCN's contact and reaction with the surface-active site from the ternary NFO/SnO_2_/CeO_2_ nanocomposite.^69,70^

A photocatalytic recycling test was conducted to assess the stability and reusability of the ternary NFO/SnO_2_/CeO_2_ nanocomposite. The composite was centrifuged, cleaned, and dried for use after every run.

**Fig. 12:**
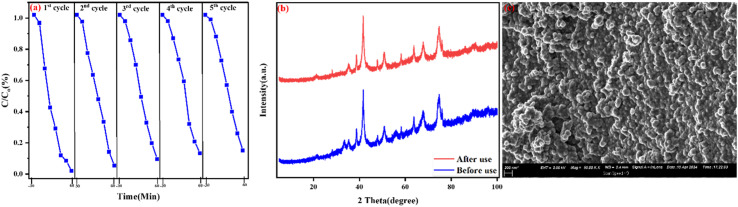
(a) Cycling test for the photocatalytic degradation of TCN (b) XRD patterns of the fresh and used and (c) SEM image of the NFO/SnO_2_/CeO_2_ nanocomposite.

The five-cycle photocatalytic activity demonstrates the good stability and reusability of the ternary nanocomposites, achieving 85.18% degradation and a 12.74% reduction in degradation between the first and last cycle. The consistency in the nanocomposite's ability across all five cycles, with no significant loss in activity, confirms the structural robustness and photocorrosion resistance of the composites under visible light irradiation, as shown in Fig. 11a. The recycled NFO/SnO_2_/CeO_2_ nanocomposite's XRD pattern and SEM image showed no discernible changes when compared to the nanocomposite before use (Fig. 12b and c), which provides credence to the excellent stability and reusability of the synthesized ternary NFO/SnO_2_/CeO_2_ nanocomposite, thereby making the nanocomposite's potential for long-term practical applications in sustainable environmental remediation.

### 3.4. Photodegradation mechanism

**Fig. 13:**
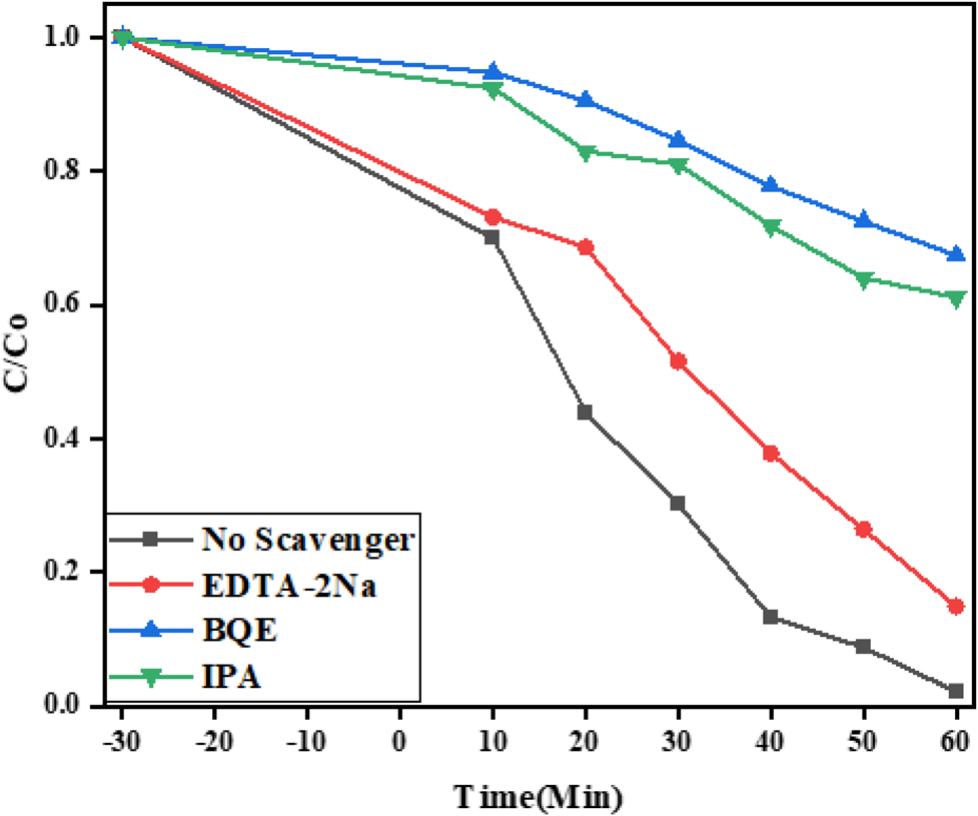
Free radical determination study of the ternary NFO/SnO_2_/CeO_2_ nanocomposite.

Reactive oxygen species (ROS) like ˙O_2_^−^, h^+^ and ˙OH are recognized to be important in photocatalytic processes.^71^ Free radical trapping studies were performed to determine the actual ROS in this process. *p*-Benzoquinone (BQE) captured the ˙O_2_^−^ ROS, ethylenediaminetetraacetate salt (EDTA-2Na) captured the h^+^, and isopropyl alcohol (IPA) captured the ˙OH. When no scavengers were present, more than 90% of the TCN was degraded, as shown in Fig. 13. Nevertheless, the TCN photodegradation was only slightly affected by the addition of Na-EDTA, suggesting that electrons and hydroxyl radicals were also important reactive species for the tetracycline degradation. Superoxide anion and hydroxyl radicals (˙O_2_^−^ and ˙OH) were the most active species in the breakdown of tetracycline, as demonstrated by the significant breakdown in TCN degradation caused by BQE and IPA.

To better understand the electron–hole migration and charge separation mechanism, the conduction band (CB) and valence band (VB) edge positions of the pristine semiconductors—NFO, SnO_2_, and CeO_2_—were calculated using the electronegativity approach (Butler & Ginley equations):^72^

9 *E*_VB_ = *χ* − *E*^e^ + 0.5*E*_g_

10 *E*_CB_ = *E*_VB_ − *E*_g_

The band edge positions of pristine NFO, SnO_2_ and CeO_2_ composites with distinct bandgap energy values at around 1.73 eV, 3.05 eV and 2.71 eV were used in calculating the valence band (VB) and conduction band (CB) energy levels.

Herein, *E*^e^ represents the energy of free electrons on the hydrogen scale which is approximately 4.5 eV while, *χ* implies the Mulliken absolute electronegativity of atom components in the specified semiconductor (such as; NFO = *χ* 4.66 eV; SnO_2_ = *χ* ∼6.33 eV; and CeO_2_ = *χ* ∼5.56 eV).^43,68^ With *E*_g_ representing the bandgap energy position of the synthesised composites, *E*_CB_ and *E*_VB_ stand for the edge potentials of the conduction and valence band potential of the composites. Using the equation given in the *E*_CB_ and *E*_VB_, values were calculated to be: NFO is at −0.71 eV and +1.02 eV; SnO_2_ is at +0.20 eV and +3.25 eV; CeO_2_ is at −0.29 eV and +2.42 eV *vs.* the normalised hydrogen electrode (NHE).

**Fig. 14:**
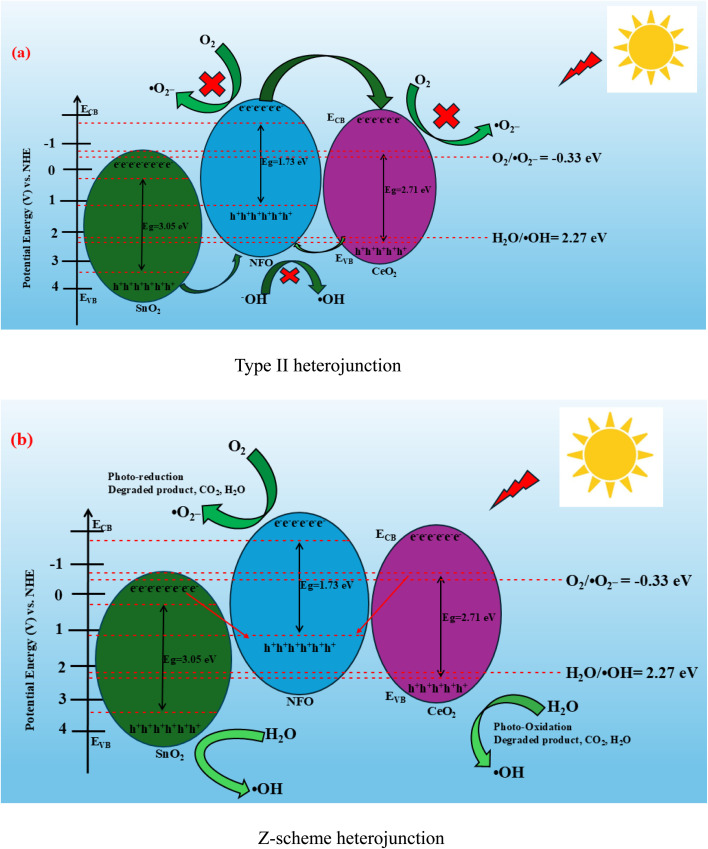
(a and b) Schematic illustration of the mechanism for the photocatalytic degradation of TCN under visible light irradiation over NFO/SnO_2_/CeO_2_ nanocomposite.

Under visible light irradiation, all three semiconductors can absorb photons and generate photogenerated e^−^/h^+^ pairs; however, the type II heterojunction explanation is deemed unreasonable, as shown in Fig. 14a, and as such, a Z-scheme charge transfer mechanism is proposed based on band alignment. In this configuration, electrons in the conduction bands of SnO_2_ and CeO_2_ migrate and recombine with holes in the valence band of NFO due to the potential differences. This spatial separation of charges retains high-energy electrons in the CB of NFO and holes in the VB of SnO_2_, leading to enhanced charge separation and suppressed recombination. The CB of NFO (−0.71 eV) is more negative than the reduction potential of O_2_/˙O_2_^−^ (−0.33 eV), enabling the reduction of adsorbed oxygen molecules to ˙O_2_^−^. Simultaneously, the VB of SnO_2_ (+3.25 eV) is more positive than the oxidation potential of H_2_O/˙OH (+2.27 eV), facilitating the generation of ˙OH radicals through the oxidation of surface hydroxyl groups or water molecules.

This effective Z-scheme charge transfer pathway significantly enhances the production of ROS while maintaining the strong redox potential required for effective TCN degradation. The combined effect of efficient light absorption, enhanced charge separation, and a high surface area, due to the ternary structure, contributes to the superior photocatalytic performance observed. The proposed mechanism is consistent with the outcomes of the radical scavenging experiments and is schematically illustrated in Fig. 14b. Overall, the integration of SnO_2_ and CeO_2_ with NFO to form a ternary heterostructure not only broadens the visible light absorption range but also facilitates efficient electron–hole separation and ROS generation, thereby enabling effective and robust degradation of TCN under visible light.

11 NFO, SnO_2_, CeO_2_ + *hν* → e^−^ + h^+^

12 e_SnO_2__^−^ + h_NFO_^+^ → recombination

13 e_CeO_2__^−^ + h_NFO_^+^ → recombination

14 e_NFO_^−^ + O_2_ → O_2_^−^

15 h_SnO_2__^+^ + H_2_O → ˙OH + H^+^

16 ˙O_2_^−^/˙OH + TCN → intermediate products → CO_2_ + H_2_O + other small molecules

## 4. Conclusion

An innovative and efficient visible-light-driven ternary nanocomposite NFO/SnO_2_/CeO_2_ was successfully prepared *via* a simple hydrothermal synthesis method aimed at enhancing the photocatalytic activity performance and energy storage potential of NFO through the formation of a double heterojunction with SnO_2_ and CeO_2_ semiconductors. The crystalline structure, surface morphology, optical properties, and textural characteristics of the ternary NFO/SnO_2_/CeO_2_ nanocomposite were extensively investigated. Notably, under visible light irradiation, the nanocomposite's photocatalytic activity for TCN degradation reached 97.93%. The improved performance of the synthesised ternary nanocomposite can be attributed to the synergistic effects of SnO_2_ and CeO_2_ nanoparticles on the surface of NFO, which enhances their capacity for visible light absorption with improved charge separation of the photogenerated charge carriers (h^+^–e^−^) due to the increased surface area. All synthesised materials showed great electrochemical performance as an electrode for supercapacitor applications, with the fabricated NFO_2_/SnO_2_/CeO_2_, ternary nanocomposite exhibiting a good specific capacity of 106.7 mA h g^−1^ at a specific current of 1 A g^−1^ within a wide stable working potential window of 0.0–0.45 V *vs.* Ag/AgCl. It achieved an excellent coulombic efficiency of 98.7% with good capacity retention of 74.5% up to 4000 galvanostatic charge/discharge cycles at 10 A g^−1^. After five consecutive cycles, the nanocomposite maintains its effectiveness, shows good durability, is easily separated from water, and demonstrates amazing stability and outstanding reusability. The trapping experiment demonstrated that superoxide and hydroxyl radicals played a crucial role in the oxidation of TCN to CO_2_ and water. The synthesised ternary nanocomposite demonstrates significant potential for removing organic contaminants from wastewater, and its exceptional stability and reusability can be useful for future environmental remediation applications. Future work will prioritise pilot-scale reactor designs with immobilised catalysts and real wastewater testing to bridge lab-to-field gaps in kinetics, fouling, and separation. Cost reduction, hybrid AOP integration, and AI-driven optimisation will be critical for achieving sustainable, large-scale TCN elimination in WWTPs.

## Conflicts of interest

The authors declare no conflict of interest, financial or otherwise.

## Supplementary Material

RA-015-D5RA07855E-s001

## Data Availability

The data supporting the research's findings are available from the corresponding author upon reasonable request. The datasets generated during and/or analyzed during the current study are not publicly available but are available from the corresponding author on reasonable request. Additionally, supplementary data and materials have been included. Supplementary information is available. See DOI: https://doi.org/10.1039/d5ra07855e.
