# Supplementary material for: Surface engineered NiFe2O4/SnO2/CeO2 ternary heterojunction for dual applications in photocatalytic water treatment and supercapacitors
Source: RSC Adv. 2025 Nov 13;15(52):44392–409. doi: 10.1039/d5ra07855e (PMC12614312; doi:10.1039/d5ra07855e)
Supplement: RA-015-D5RA07855E-s001 [file RA-015-D5RA07855E-s001.pdf]

Supplementary Information (SI) for RSC Advances.
This journal is © The Royal Society of Chemistry 2025

Surface Engineered $NiFe_2O_4/SnO_2/CeO_2$ Ternary Heterojunction for Dual Applications in Photocatalytic Water Treatment and Supercapacitors

Adewumi O. Oluwole[a], Samba Sarr[a], Tunde L. Yusuf[b], Shepherd M. Tichapondwa[a], Michael O. Daramola[a], Samuel A. Iwarere[a*]

[a] Department of Chemical Engineering, Faculty of Engineering, Built Environment and Information Technology, University of Pretoria, Hatfield, Pretoria 0002, South Africa

[b] Department of Chemistry, Faculty of Natural and Agricultural Sciences, University of Pretoria, Hatfield, Pretoria 0002, South Africa

*Corresponding author. Tel: +27-12-420-3092. E-mail: samuel.iwarere@up.ac.za (Samuel Iwarere)

Text S1: Characterization

Powder X-ray diffraction (XRD) measurements using a PANalytical X'Pert Pro powder X-ray diffractometer in θ–θ configuration and Cu-Kα radiation (λ =1.789 Å) were used to investigate the structure and crystallinity of the synthesized nanocomposites. A PerkinElmer Spectrum One spectrometer was used to record the FTIR spectra of the photocatalyst samples using the KBr pellet approach. To examine the morphologies and elemental composition, field emission scanning electron microscopy (FESEM, JSM-6400) fitted with an EDS analyzer was utilized. A Thermo Scientific ESCALAB 250Xi spectrometer equipped with a monochromatic Al-Kα X-ray source (1486.7 eV) at 150 W was used to perform X-ray photoelectron spectroscopy (XPS) on the ternary nanocomposite material to assess its surface valence, elemental compositions, and electronic structure. The specific surface area and pore distribution of the nanocomposites were assessed using the Brunauer–Emmett–Teller (BET) micrometrics Tristar II 3020 Version 3.02 system. To evaluate the nanocomposites' ability to absorb light, a UV-vis spectrophotometer (Agilent Technologies Cary 60 UV-vis, Malaysia) was utilized.

Text S2: Electrochemical Procedures

In this study, the electrodes used were fabricated by mixing the as-prepared samples as active materials, acetylene carbon black (ACB) as the conductive additive and polyvinylidene fluoride (PVDF) as a binder in a mass ratio (in percentage) of 80:10:10 respectively, in an agate mortar with the dropwise addition of N-methyl pyrrolidone (NMP) to make a homogenous slurry. The

resulting slurry was then pasted onto rectangular (1*2 cm^2) clean nickel form adopted as a current collector to fabricate electrodes for three-electrode measurements. The electrodes were then dried at 60 °C in an electric oven overnight. Afterwards, the supercapacitor performances of the fabricated electrodes were obtained using a Bio-Logic VMP-300 potentiostat (Knoxville, TN, USA), then EC-Lab V11.33 software (Edmonton, AB, Canada) was used to explore the data.

Text S3: Reactor set-up for Photocatalytic procedures

As previously described by AO Oluwole*, et al.* [1], the photoreactor setup was made up of a wooden box consisting of four Philips 18 W fluorescent daylight lamps to serve as the source of visible light irradiation, each emitting a luminous flux of 1200 lm. Aluminium foil paper was used to line the inside of the wooden box so that light could be reflected and distributed evenly. Three batches of the slurry solution were continually stirred using three magnetic stirrers that were positioned inside the box approximately 10 cm from the lights. The light intensity in the photocatalytic process was calculated using the fluorescent lamps' wattage and the distance between the reactor and the lamp, yielding 142.86 W/m^2. Conversely, a thermometer positioned within the wooden box was used to measure the reaction setup's temperature, which came out to be 30 ◦C (±5).

The photocatalytic activities of the synthesized composites $NiFe_2O_4$, SnO_2, CeO_2, $NiFe_2O_4/SnO_2$, $NiFe_2O_4/CeO_2$ and $NiFe_2O_4/SnO_2/CeO_2$ were assessed using TCN, a common antibiotic. In a nutshell, 50 mg of catalyst was added to 100 mL of TCN solution (30 mg/L, pH 5.5). To achieve the absorption–desorption balance, the suspension was continuously swirled for 30 minutes in the dark prior to illumination. The photocatalyst–pollutant suspension was then illuminated at room temperature using a commercial fluorescent lamp. 5 mL aliquots were taken at predetermined intervals during the exposure to visible light, centrifuged and filtered through a 0.45 µm membrane filter to eliminate any remaining catalyst particles. A UV–vis spectrophotometer (Biochrom, Cambridge, UK) was used to evaluate the filtrates at the absorption wavelength of 357 nm.

The degree of TCN removal was quantitatively evaluated using a Shimadzu TOC-V analyzer, prior to and following the photocatalytic process.

The following formula was used to determine the photocatalyst's photocatalytic efficiency for pollutant degradation:

$$\text{Removal efficiency} = \frac{C_0 - C}{C_0} \times 100\% \quad (1)$$

C0 stands for the concentration of the pollutants at the time (t_0) (initial time) before treatment, while C is the concentration value at the final time after treatment.

Table S.1: BET surface areas, pore volumes, and pore size diameter of the materials

Material	Surface area ($m^2\ g^{-1}$)	Pore Volume ($cm^3\ g^{-1}$)	Pore Diameter (nm)	Crystalline size (nm)
NFO	26.82	0.00022	27.56	36.49
SnO_2	11.77	0.00078	35.46	21.24
CeO_2	41.58	0.00085	35.20	16.05
NFO/SnO_2	38.71	0.00090	36.45	27.36
NFO/CeO_2	51.93	0.0016	36.27	20.72
NFO/SnO_2/CeO_2	72.99	0.0077	38.99	10.81

Figure S.1: SEM images of the as-prepared samples: (a) $NiFe_2O_4$, (b) SnO_2, (c) CeO_2, (d) $NiFe_2O_4/SnO_2$, (e) $NiFe_2O_4/CeO_2$ and (f) nanocomposite $NiFe_2O_4/SnO_2/CeO_2$

(a)

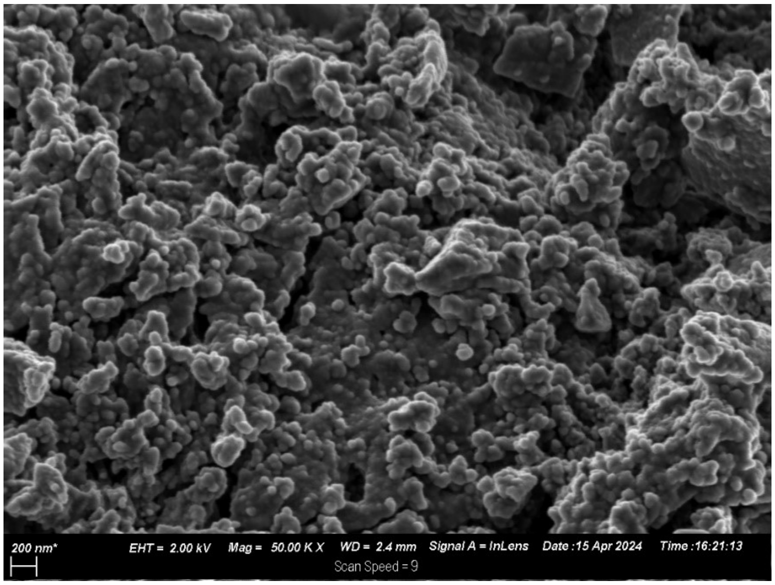

(b)

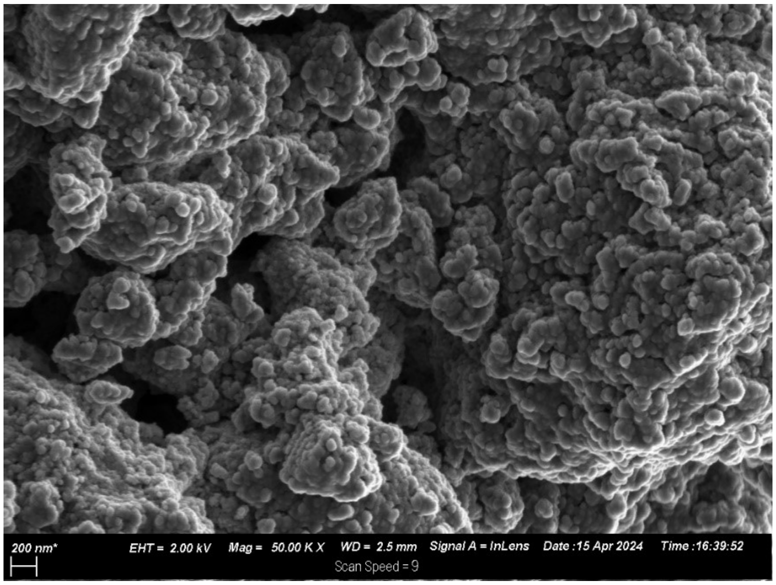

(c)

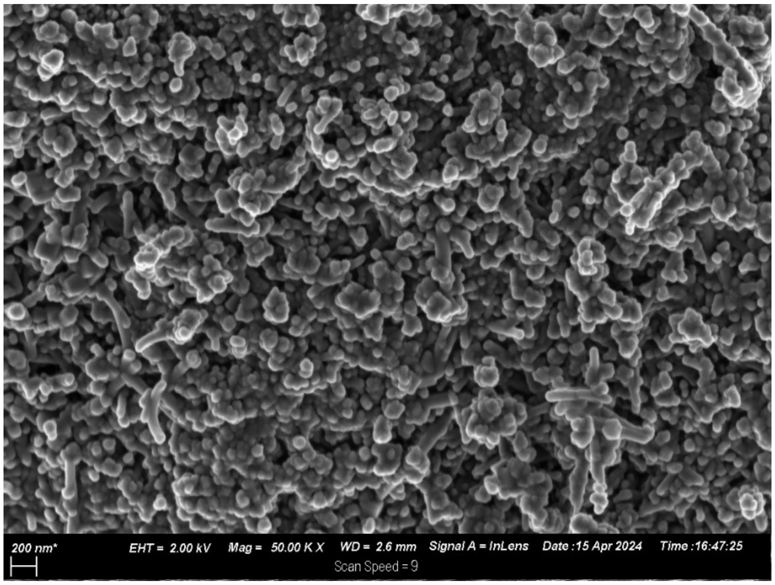
200 nm*
EHT = 2.00 kV
Mag = 50.00 K X
WD = 2.6 mm
Signal A = InLens
Date :15 Apr 2024
Time :16:47:25
Scan Speed = 9

(d)

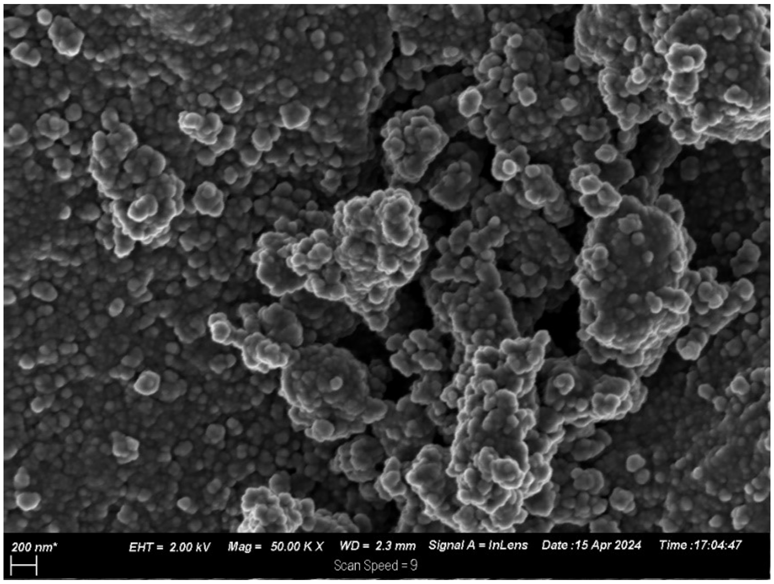
200 nm*
EHT = 2.00 kV
Mag = 50.00 K X
WD = 2.3 mm
Signal A = InLens
Date :15 Apr 2024
Time :17:04:47
Scan Speed = 9

(e)

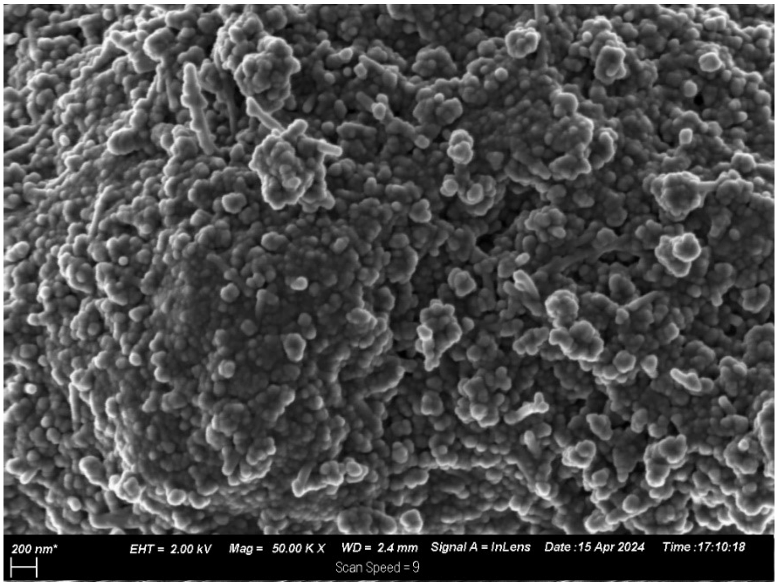
200 nm*
EHT = 2.00 kV
Mag = 50.00 K X
WD = 2.4 mm
Signal A = InLens
Date :15 Apr 2024
Time :17:10:18
Scan Speed = 9

(f)

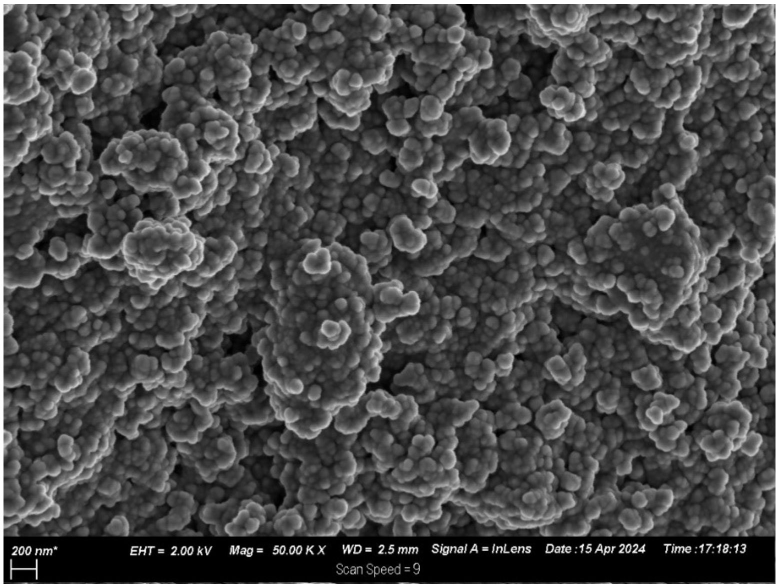
200 nm*
EHT = 2.00 kV
Mag = 50.00 K X
WD = 2.5 mm
Signal A = InLens
Date :15 Apr 2024
Time :17:18:13
Scan Speed = 9

Figure S.2. Expanded energy band gap plots for the synthesized nanocomposites

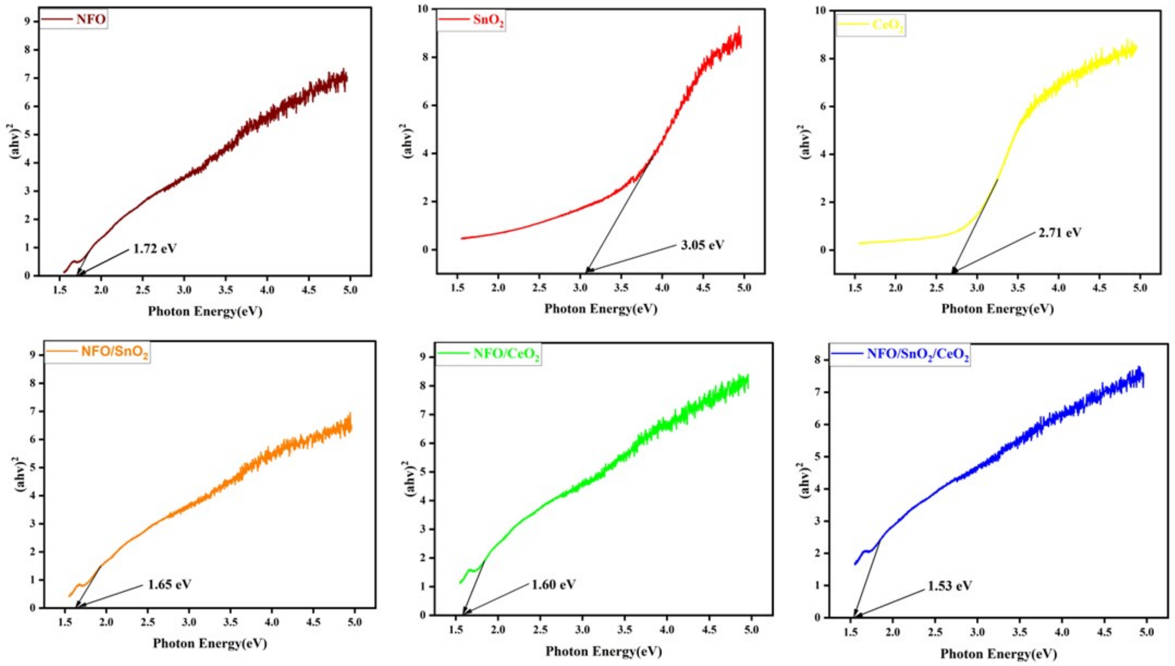

Table S.2. Comparison of photocatalytic performance with other previously reported photocatalysts for the degradation of TCN in recent years.

Nanocomposites	Amount (mg/L)	Dosage (mg)	Light source (Lamp)	Photocatalytic efficiency	References
$NiFe_2O_4/CeO_2/GO$	20	50	300 W Xenon	95 % in 90 min	[2]
$ZnO/NiO/g\text{-}C_3N_4$	20	50	500 W Halogen	91 % in 60 min	[3]
$Fe_3O_4/BiVO_4/CdS$	10	100	300 W Xenon	87 % in 90 min	[4]
$g\text{-}C_3N_4$/ultrathin MoS_2	10	10	250 W metal halide	96 % in 4 h	[5]
$Cu\text{-}Fe_2O_3/Ni\text{-}ZnO$	10	40	Xenon lamp (light intensity of 300 mW/cm^2	94 % in 120 min	[6]
Fe_3O_4/ZnO	50	40	120-W PAR38 warm light bulb	75 % in 120 min	[7]
$BiOCl/Bi_2WO_6$	100	20	350 W xenon	94 % in 60 min	[8]
$Bi/\alpha\text{-}Bi_2O_3/g\text{-}C_3N_4$	10	10	300 W Xenon	91 % in 180 min	[9]
$BiOCl@CeO_2$	50	10	300 W Xenon	90 % in 120 min	[10]
$NFO/SnO_2/CeO_2$	30	50	72 W fluorescent daylight	97.92 % in 60 min	This study

Figure S.3: SEM image of the NFO/SnO_2/CeO_2 nanocomposite after 5-cycle runs for the photodegradation of TCN

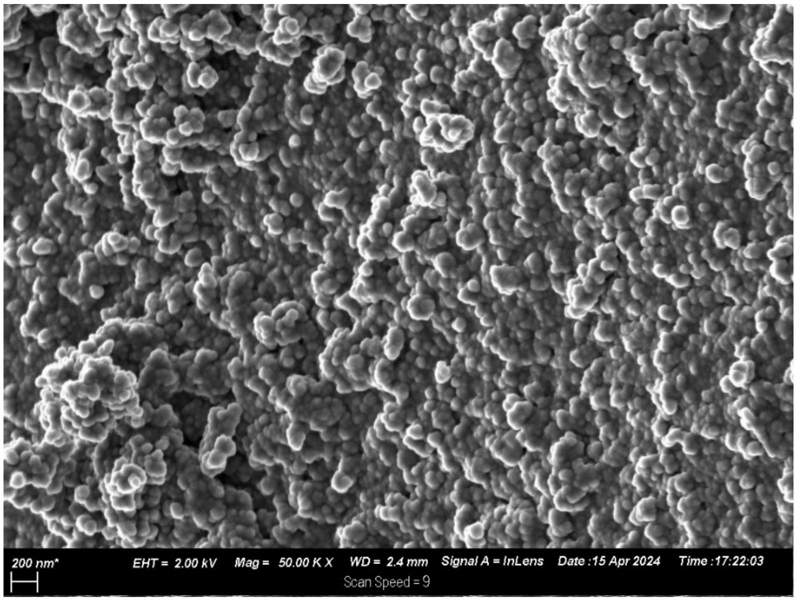

References

1. Oluwole AO, Yusuf TL, Tichapondwa SM, Daramola MO, Iwarere SA: **Enhanced photocatalytic efficiency of a novel GO/Bi2SO5/AgBr ternary heterojunction for the degradation of tetracycline and rhodamine B**. *J Environ Chem Eng* 2025:116777.
2. Nawaz R, Aziz MH, Asif M, Noor F, Aligayev A, Ali SM, Alam M, Papanikolaou S, Huang Q: **Evaluation of tetracycline photocatalytic degradation using NiFe2O4/CeO2/GO nanocomposite for environmental remediation: In silico molecular docking, antibacterial performance, degradation pathways, and DFT calculations**. *Sep Purif Technol* 2024, **351**:128074.
3. Dineshbabu N, Jayaprakash R, Karuppasamy P, Arun T, Vijaya JJ, Nimshi RE, Pandian MS, Packiam SM, Ramasamy P: **Investigation on Tetracycline degradation and bactericidal properties of binary and ternary ZnO/NiO/g-C3N4 composites prepared by a facile co-precipitation method**. *J Environ Chem Eng* 2022, **10**(3):107368.
4. Xu G, Du M, Li T, Guan Y, Guo C: **Facile synthesis of magnetically retrievable Fe3O4/BiVO4/CdS heterojunction composite for enhanced photocatalytic degradation of tetracycline under visible light**. *Sep Purif Technol* 2021, **275**:119157.
5. Lu X, Jin Y, Zhang X, Xu G, Wang D, Lv J, Zheng Z, Wu Y: **Controllable synthesis of graphitic C 3 N 4/ultrathin MoS 2 nanosheet hybrid nanostructures with enhanced photocatalytic performance**. *Dalton Transactions* 2016, **45**(39):15406-15414.
6. Li Y, Liu K, Zhang J, Yang J, Huang Y, Tong Y: **Engineering the band-edge of Fe2O3/ZnO nanoplates via separate dual cation incorporation for efficient photocatalytic performance**. *Ind Eng Chem Res* 2020, **59**(42):18865-18872.
7. Sabar S, Jamil AM, Zhao Y, Yusof ENM, Schneider R, Mohamed AR, Kuwahara Y, Mori K, Yamashita H: **Construction of Fe 3 O 4/ZnO heterostructure photocatalyst derived from Fe-doped ZIF-8 for enhanced photocatalytic degradation of tetracycline and hydrogen peroxide production**. *New J Chem* 2025.
8. Zhang H, Zhu Z, Huang Y, Yu J, Li M: **Construction of Z-Scheme Heterojunction BiOCl/Bi2WO6 for Visible-Light Photocatalytic Degradation of Tetracycline Hydrochloride**. *Separations* 2025, **12**(5):111.
9. Chen D, Wu S, Fang J, Lu S, Zhou G, Feng W, Yang F, Chen Y, Fang Z: **A nanosheet-like α-Bi2O3/g-C3N4 heterostructure modified by plasmonic metallic Bi and oxygen vacancies with high photodegradation activity of organic pollutants**. *Sep Purif Technol* 2018, **193**:232-241.
10. Wang H, Liao B, Lu T, Ai Y, Liu G: **Enhanced visible-light photocatalytic degradation of tetracycline by a novel hollow BiOCl@ CeO2 heterostructured microspheres: Structural characterization and reaction mechanism**. *J Hazard Mater* 2020, **385**:121552.
